# Supplementary material for: Broad substrate scope C-C oxidation in cyclodipeptides catalysed by a flavin-dependent filament
Source: Nat Commun. 2025 Jan 24;16:995. doi: 10.1038/s41467-025-56127-y (PMC11760959; doi:10.1038/s41467-025-56127-y)
Supplement: Supplementary file 1 — Supplementary Information [file 41467_2025_56127_MOESM1_ESM.pdf]

Broad substrate scope C-C oxidation in cyclodipeptides catalysed by a flavin-dependent filament

Emmajay Sutherland[1] †, Christopher J. Harding[1], Tancrede Martin Y Du Monceau De Bergendal[1], Gordon J. Florence[2], Katrin Ackermann[2], Bela E. Bode[2], Silvia Synowksy[3], Ramasubramanian Sundaramoorthy[4], and Clarissa Melo Czekster[1]**

1 University of St Andrews, School of biology, North Haugh, Biomolecular Sciences building, KY16 9ST

2 University of St Andrews, EaStCHEM School of Chemistry, North Haugh, Purdie building, KY16 9ST

3 University of St Andrews, BSRC Mass Spectrometry and Proteomics Facility, North Haugh, Biomolecular Sciences building, St Andrews, KY16 9ST, UK

4 Laboratory of Chromatin Structure and Function, MCDB, School of Life Sciences, University of Dundee, Dundee, DD1 5EH, UK

***To whom correspondence should be addressed:**

Clarissa Melo Czekster - School of Biology, University of St Andrews, Biomedical Sciences Research Complex, St Andrews, Fife KY16 9ST, U.K. Email: cmc27@st-andrews.ac.uk

Ramasubramanian Sundaramoorthy - Laboratory of Chromatin Structure and Function, MCDB, School of Life Sciences, University of Dundee, Dundee, DD1 5EH, UK. Email: R.Z.Sundaramoorthy@dundee.ac.uk

Supplementary Methods 4
*Ndas*CDO DNA sequences 4
*Ndas*1146 4
Ndas1147 4
NdasCDO and mutant-NdasCDO protein sequences 4
*Ndas*1146 4
*Ndas*1147 4
*Ndas*1146-S58A 4
*Ndas*1146 2-17 truncation 5
*Ndas*1146 2-39 truncation 5
Assay conditions 5
Docking of CDP substrates 6
Supplementary Figures 7
Supplementary Figure 1: Genomic context for NdasCDO 8
Supplementary Figure 2: FMN cofactor is covalently bound to *Ndas*CDO. 9
Supplementary Figure 3: UV difference spectra upon CDP oxidation 11
Supplementary Figure 4: Temperature rate profiles. 13
Supplementary Figure 5: Overview of cryo-EM structure. 14
Supplementary Figure 6: Details on the FMN binding site and overall structure of A and B subunits 15
Supplementary Figure 7: Comparison of *Ndas*CDO to AlbAB[4]. 16
Supplementary Figure 8: Structural alignment of *Ndas*1146 with NfsA. 17
Supplementary Figure 9: $\text{NdasCDO}_{\text{S58A}}$ kinetic analysis. 17
Supplementary Figure 10: Raw kinetic data related to Figure 5. 18
Supplementary Figure 11: LC-MS chromatograms reporting CDP oxidation 19
Supplementary Figure 12: Progress Curve for the reaction with cFP. 21
Supplementary Figure 13: Unique fragmentation products expected after oxidation of cLP. 22
Supplementary Figure 14: Docking of CDP substrates. 26
Supplementary Figure 15: Proton inventories for the NdasCDO-catalysed reaction. 27
Supplementary Figure 16: NdasCDO Viscosity studies with glycerol, sucrose and PEG-8K. 28
Supplementary Figure 17: NdasCDO plasmid map 29
Supplementary Figure 18: Data processing pipeline. 30
Supplementary Figure 19: Summary of reconstruction of Cryo-EM data. 31
Supplementary Tables 32

Supplementary Table 1: Primers for site directed mutagenesis of *Ndas*CDO 32
Supplementary Table 2: Relative viscosities (ηrel) for different concentrations of glycerol and sucrose 32
Supplementary Table 3: Kinetic parameters for NdasCDO substrates 33
Supplementary Table 4: Data collection and refinement statistics 34
Supplementary note 1: synthesis and characterization of cWS 35
Supplementary References 46

Supplementary Methods

*Ndas*CDO DNA sequences

*Ndas*1146

ATGGACACAGGTTCGAGCGAGCCGGATGCGAACCGGTGCCCCTCTCAGCGGT
CATCACACGCCCTACAGACCCTGACCACCCGCCGTGCCGTACGCGCCTTCGCC
GACCGGCCGGTGGACGACTCCCTCCTCGACCCCATGCTGGACGCCATGCTCG
CCGCCCCCTCGGCGTCCAACAAGCAGGCGTGGGCCTTCGTCGCCGTCCGCGA
GCGGCGGGCGCTGAGGCTGCTGCGCGCCTTCTCCCCCGGAATCATCGAACTC
CCGCCCCTGGTCGTGGCGGCCTGCTTCGACCGCTCCCGTGCCGTGGGGGGCT
CAGGCAACTCCACGGACTCCGGGGACTCCTGGGACGAGGGCATGCTCTGCGT
CGCGATGGCGGTGGAGAACCTCCTCCTGGCGGCCCACTGCCTGGGGCTGGGC
GGATGCCCGTCCGGGAGCTTTCGGAGGGGCCCCGTCCGCAGGCTCCTGGGC
CTGCCCGACCACCTGGAACCCCTGCTCCTGGTTCCGATCGGGCACCCCGCCC
GGCCACTCGCACCCGCACCCCGACGAGACCGGAACGAGGTGGTCAGCCATGA
GCGCTGGGGAACC

Ndas1147

ATGAGCGCTGGGGAACCTGAGGTCCGACAGGTCGGCGAGGAACTCCTCCTGC
TCGCCGCCTACCTGCTCAGCAGCGGCCGCGGCCTGCTGGACGAGCCACGGCA
GTACGGCACGTTCCGCTGCCTGGACGCCGCCCGGCGCGTCCTCGCCCTCGCG
GCCGGAACCGGCCCGCACCACCCCGAACTCGACGCCCTGCGCGGTCGGATG
GACGACGTCATGTGCGGGCCGATGGGCGACCACGAACTGGACACCCTGCTCG
ACCAGATGTGCGAGCGGCTGGCAACCGTCCTGGAGGATCCCGATGTCATCTCC
GACTGA

NdasCDO and mutant-NdasCDO protein sequences (additional residues on the C-terminus+histag highlighted yellow:

*Ndas*1146

MDTGSSEPDANRCPSQRSSHALQTLTTRRAVRAFADRPVDDSLLDPMLDAMLAAP
SASNKQAWAFVAVRERRALRLLRAFSPGIIELPPLVVAACFDRSRAVGGSGNSTDS
GDSWDEGMLCVAMAVENLLLAAHCLGLGGCPSGSFRRGPVRRLLGLPDHLEPLLL
VPIGHPARPLAPAPRRDRNEVVSHERWGTGSSHHHHHH

*Ndas*1147

MSAGEPEVRQVGEELLLLAAYLLSSGRGLLDEPRQYGTFRCLDAARRVLALAAGT
GPHHPELDALRGRMDDVMCGPMGDHELDTLLDQMCERLATVLEDPDVISD

*Ndas*1146-S58A

MDTGSSEPDANRCPSQRSSHALQTLTTRRAVRAFADRPVDDSLLDPMLDAMLAAP
SAANKQAWAFVAVRERRALRLLRAFSPGIIELPPLVVAACFDRSRAVGGSGNSTDS
GDSWDEGMLCVAMAVENLLLAAHCLGLGGCPSGSFRRGPVRRLLGLPDHLEPLLL
VPIGHPARPLAPAPRRDRNEVVSHERWGTGSSHHHHHH

*Ndas*1146 2-17 truncation

MSSHALQTLTTRRAVRAFADRPVDDSLLDPMLDAMLAAPSASNKQAWAFVAVRERRALRLLRAFSPGIIELPPLVVAACFDRSRAVGGSGNSTDSGDSWDEGMLCVAMAVENLLLAAHCLGLGGCPSGSFRRGPVRRLLGLPDHLEPLLLVPIGHPARPLAPAPRRDRNEVVSHERWGTGSSHHHHHH

*Ndas*1146 2-39 truncation

MDDSLLDPMLDAMLAAPSASNKQAWAFVAVRERRALRLLRAFSPGIIELPPLVVAACFDRSRAVGGSGNSTDSGDSWDEGMLCVAMAVENLLLAAHCLGLGGCPSGSFRRGPVRRLLGLPDHLEPLLLVPIGHPARPLAPAPRRDRNEVVSHERWGTGSSHHHHHH

Assay conditions – Prior work indicated a direct absorbance change occurs upon cyclic dipeptide oxidation. We expanded this direct assay with multiple CDP substrates, since the nature of amino acid side chains changes the maximum absorbance observed from substrates and products. Using the difference spectra between reactants and products, we obtained a DeltaAbs value, which was employed to convert changes in absorbance into changes in concentration as a function of time. Substrates containing Phe, Tyr and Trp had significantly red-shifted spectra in comparison to substrates containing only non-aromatic groups (Leu, Pro, His). Supplementary Figure 2 summarizes all substrates tested, with their absorption spectra, and, following overnight reactions, spectra for oxidized products (identity verified by LC-MS). A complicating factor for this assay is the fact that some CDPs can undergo two oxidation events, which can alter both kinetics and spectroscopic characteristics of intermediates. Under steady-state conditions, with excess unoxidized substrates, we observed the accumulation of a singly-oxidized product before formation of a doubly oxidized CDP in the case of cFP (Supplementary Figure 8), and therefore conclude NdasCDO operates distributively, catalyzing one oxidation event to generate an intermediate, which was released and could subsequently re-bind to the enzyme for a second oxidation event.

Data analysis for pH-rate profiles – According to Cook and Cleland (page 335), when two pK_a values are close together, a different equation might be required to fit data, as below:

$$y = log\left(\frac{C}{1+\frac{pKa2}{pKa1}+\frac{10^{-pH}}{10^{-pKa1}}+\frac{10^{-pka2}}{10^{-pH}}}\right) \qquad \text{Supplementary Equation 1}$$

However, if $K_2/K_1 < 0.1$, Equation 3 can be used, as the pK_a values are distant enough to allow individual fitting. In the case of NdasCDO, K2/K1 = 0.03, and the pKa values can be individually fitted.

Docking of CDP substrates – To provide insight into substrate orientation in the binding pocket, we carried out docking experiments with the substrates tested here.

Protein target and Ligand preparation:

Our cryoEM resolved dimer of dimers NdasAB was used as protein structure target to determine cyclic dipeptide binding mode proximal to the covalently linked FMN cofactor within the dimer of dimer interface. Polar hydrogen atoms and the Kollman charges were added to the protein target NdasAB using AutodockTools. For docking boundaries box size of 12Åx12Åx16Å with 1 Å spacing were used around FMN reaction site as origin. The AutoGrid 4.2 default parameter was used in AutoGrid4 to calculate the affinity map of each atom, the desolvation map and the electrostatic map.

Initial structures of cyclic dipeptide ligands were prepared with the ligand builder module of Coot using the smiles string. The resulted cyclic dipeptide structures were geometry optimised using the ATB server.[1] Similar to the protein targets polar hydrogens and Kollman charges were added to each cyclic dipeptide ligand using AutodockTools.

Molecular docking:

Docking of each cyclic dipeptide molecule was performed using Autodock4 suite (version 4.2.6).[2] We carried out semi-flexible docking that allows the ligands to sample various conformation during docking stage whereas the protein target and its side chains remain rigid. Lamarckian Genetic Algorithm was used to search the potential conformations. For each ligand 20 sets of Genetic Algorithm run were carried out with default docking parameter set. The external grid energy was set as 1000. The max allowable initial energy was 0. During the start of the run each cyclic dipeptide translation, orientation and dihedrals were randomised. For each docking step translation was set to 2Å and quaternion and torsions were set to 5° each. The resultant docked conformations are clustered with rms tolerance of 2. The docking poses were analysed using AutodockTools. Ligplot was used to create a 2D ligand environment. Representative images were made with Pymol and ChimeraX. Binding configurations and energies can be found in Supplementary Figure 12.

Supplementary Figures

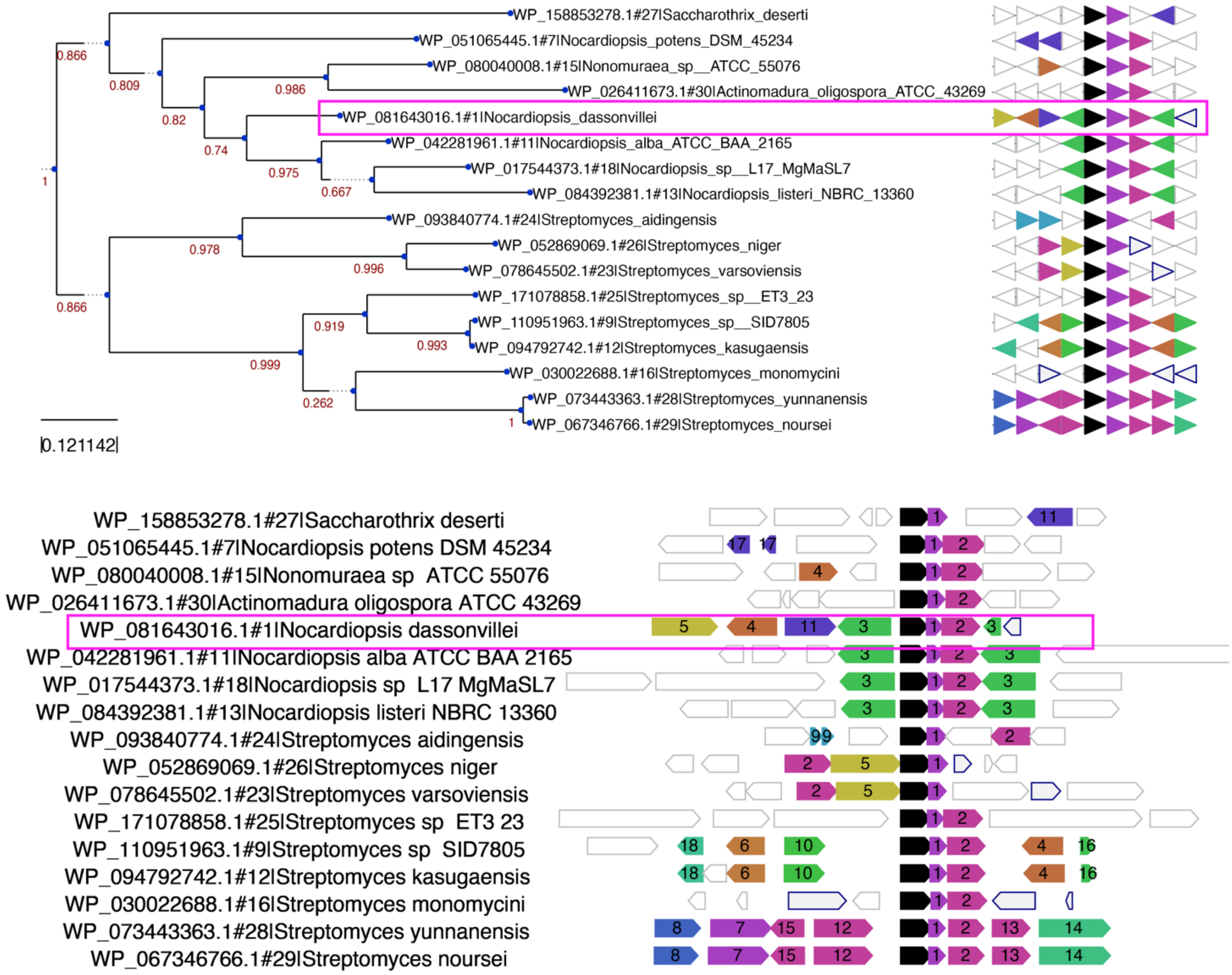

#	Example protein	Predicted function
1	WP_238413131.1	DUF6092 family protein (CDO subunit B)
2	WP_157851683.1	tRNA-dependent cyclodipeptide synthase
3	WP_232532480.1	methyltransferase
4	WP_094801803.1	SDR family NAD(P)-dependent oxidoreductase
5	WP_094801801.1	cytochrome P450
6	WP_052853172.1	MULTISPECIES: VWA domain-containing protein
7	WP_073443366.1	MULTISPECIES: cysteine desulfurase family protein
8	WP_073443368.1	thioesterase family protein

9	WP_093840776.1	DUF397 domain-containing protein
10	WP_094792743.1	HAD family hydrolase
11	WP_158853282.1	alpha/beta hydrolase
12	WP_189854609.1	tRNA 2-thiouridine(34) synthase MnmA
13	WP_073443360.1	VC0807 family protein
14	WP_073443358.1	MULTISPECIES: NADP-specific glutamate dehydrogenase
15	WP_073443723.1	MULTISPECIES: N-acetylmuramoyl-L-alanine amidase
16	WP_205380085.1	MULTISPECIES: hypothetical protein
17	WP_017593943.1	hypothetical protein
18	WP_094792745.1	helix-turn-helix domain-containing protein

Supplementary Figure 1: Genomic context for NdasCDO. Generated using Webflags (https://server.atkinson-lab.com/webflags)[3]. Top, phylogenetic tree of aligned sequences. Bottom, biosynthetic gene clusters with closest homologues of *Ndas*1146 (Uniprot: D7B1W6, shown in black).

Supplementary Figure 2: FMN cofactor is covalently bound to *Ndas*CDO.

a)

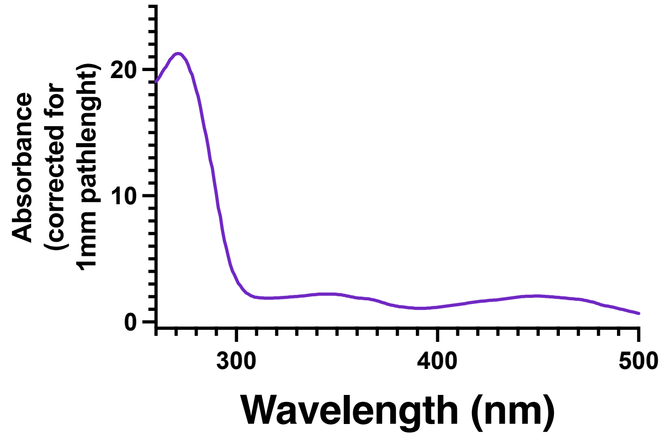

b)

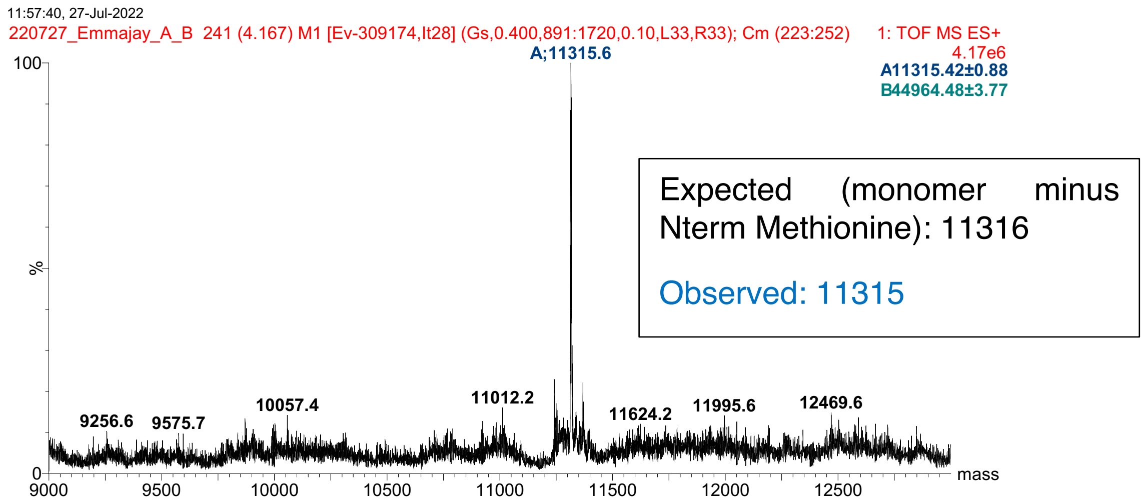

c)

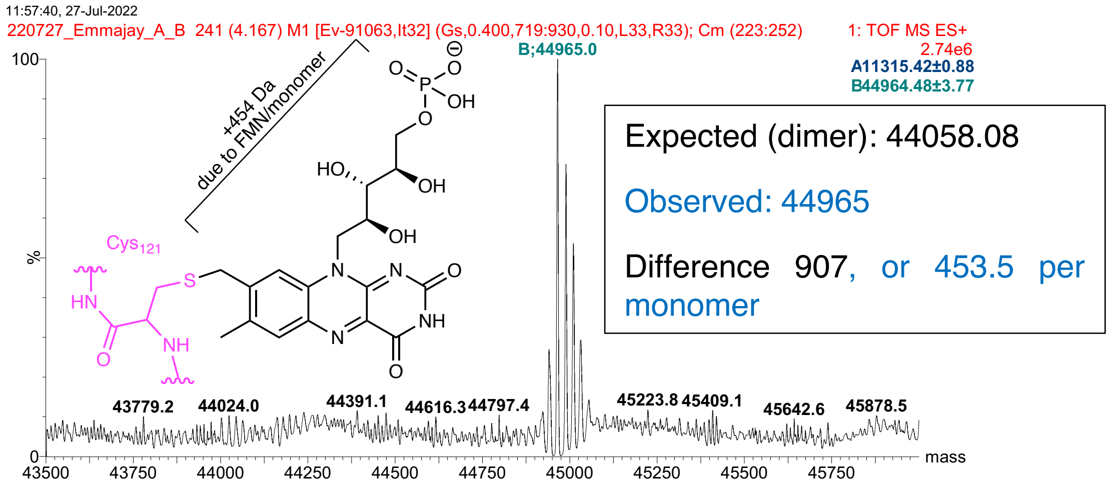

d)

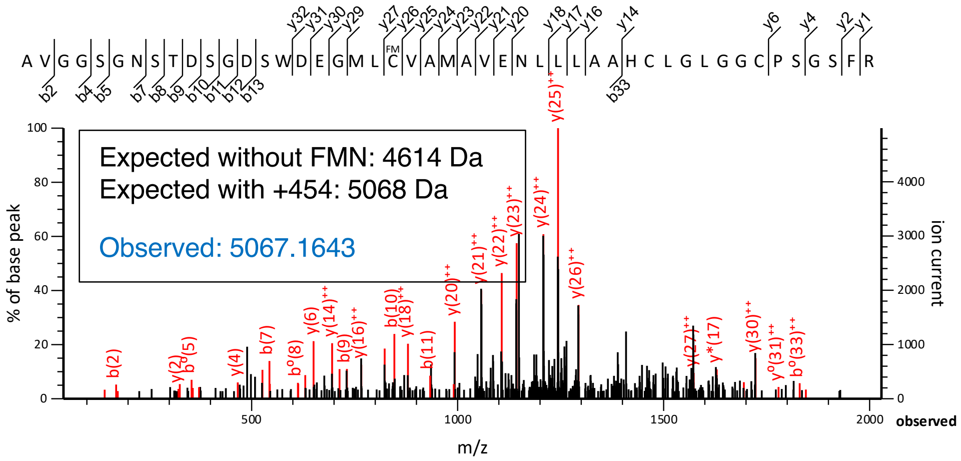

Supplementary Figure 2: FMN cofactor is covalently bound to *Ndas*CDO.

a) UV spectra of purified protein shows absorbance ~350 nm and ~450 nm as typical for flavoproteins. Protein intact mass for subunit b) shows no modifications on subunit B. c) shows subunit A presents as a dimer and is modified by the addition of one FMN per monomer. d) MS/MS spectra for peptide observed after trypsin digestion, showing FMN is covalently attached to C121 of subunit A. Peptides matched to the expected sequence and used for assignment by Mascot are shown in red.

Supplementary Figure 3: UV difference spectra upon CDP oxidation

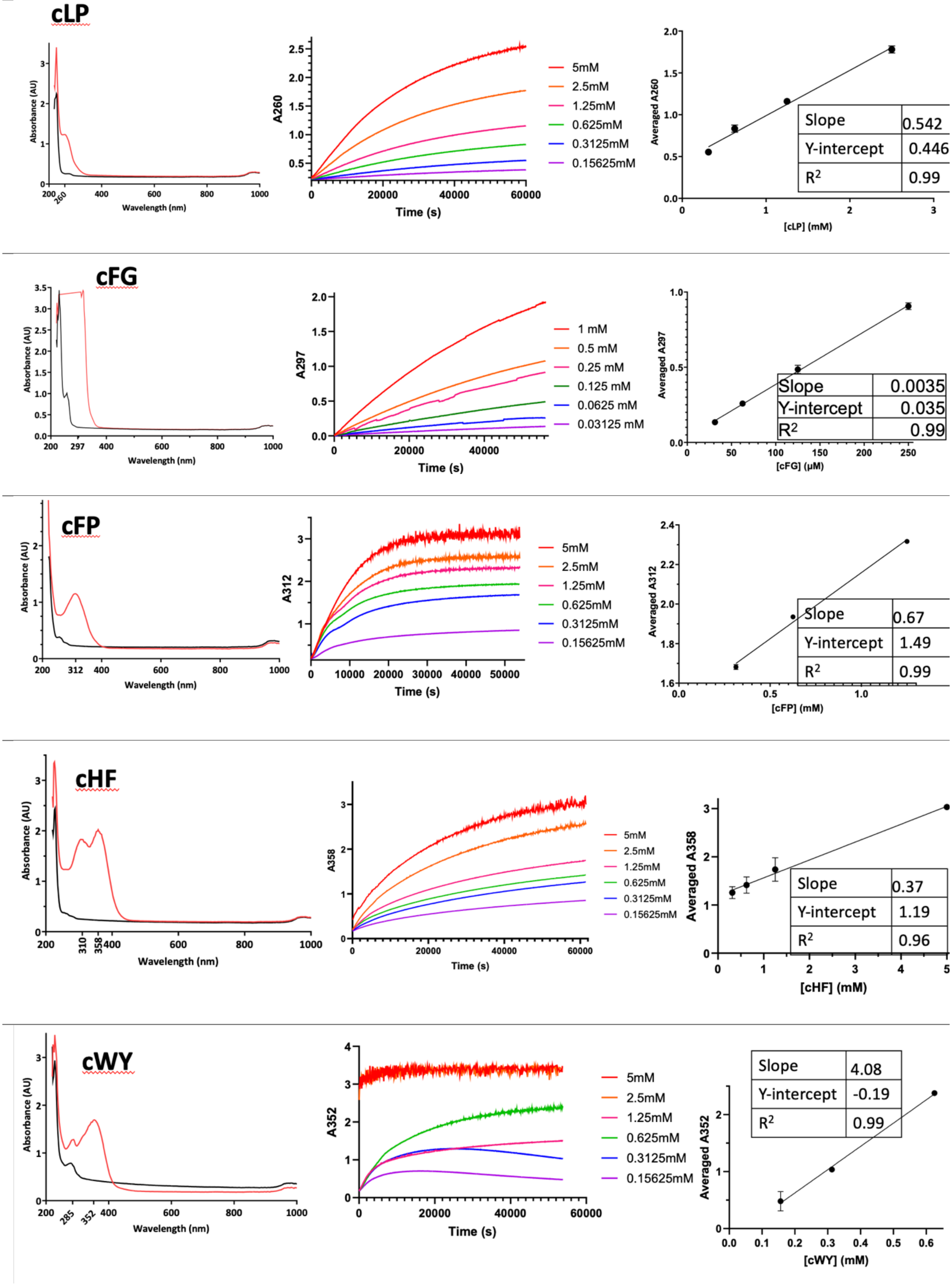

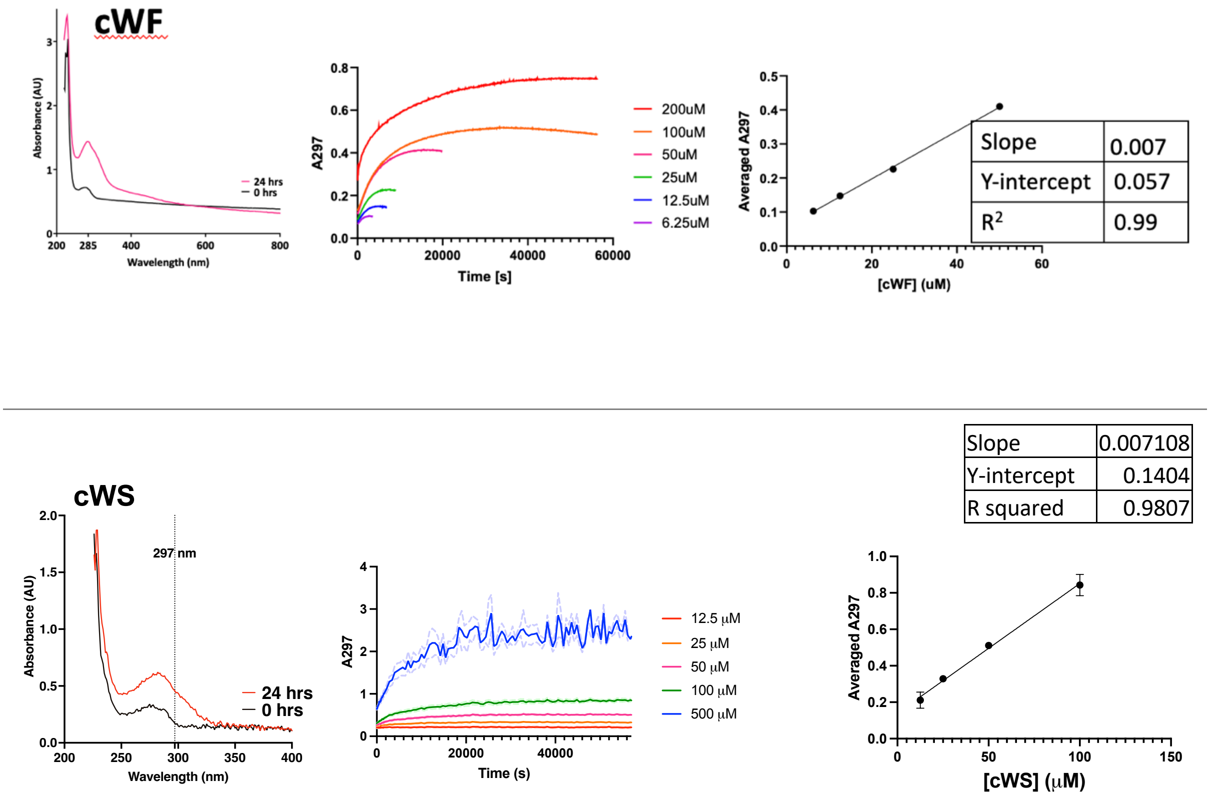

Supplementary Figure 3: UV difference spectra upon CDP oxidation. Same information for each row in the Figure . Left: difference UV spectra comparing substrates (black trace) and oxidized products after reaction reached completion (red trace); middle: progress curve for each rection until completion, endpoint UV reading was used to generate the calibration curve on the right, to obtain a relationship between absorbance change and CDP oxidation for each CDP substrate.

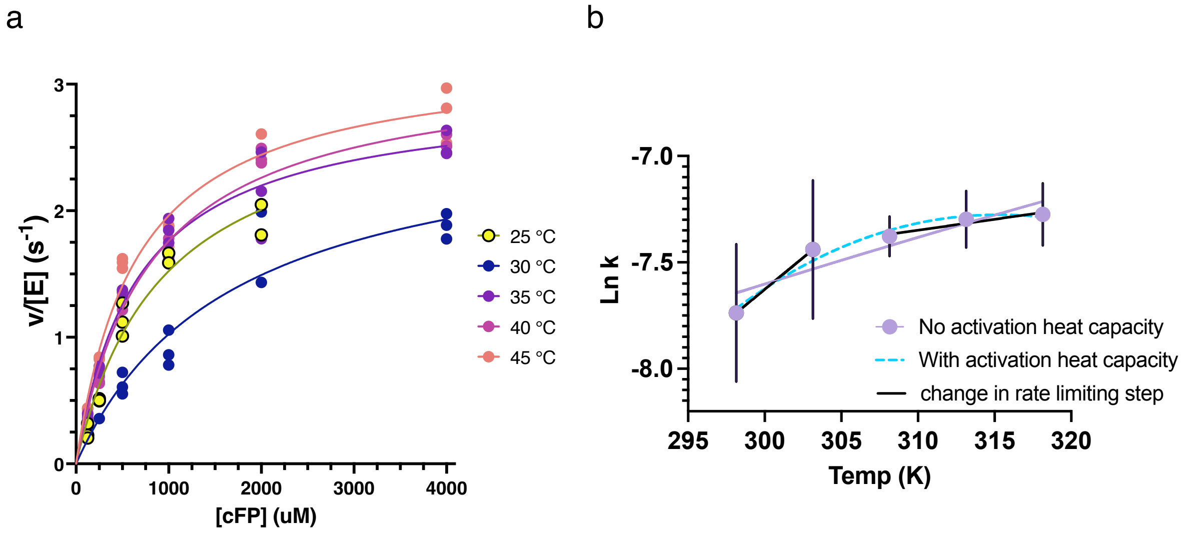

Supplementary Figure 4: Temperature rate profiles.

a) raw data for Michaelis-Menten plots at each different temperature. All data are shown. b) Comparison between fits to an Eyring Equation with and without contributions from activation heat capacity, as well as with linear fits that would occur due to changes in the rate limiting step for the reaction under study.

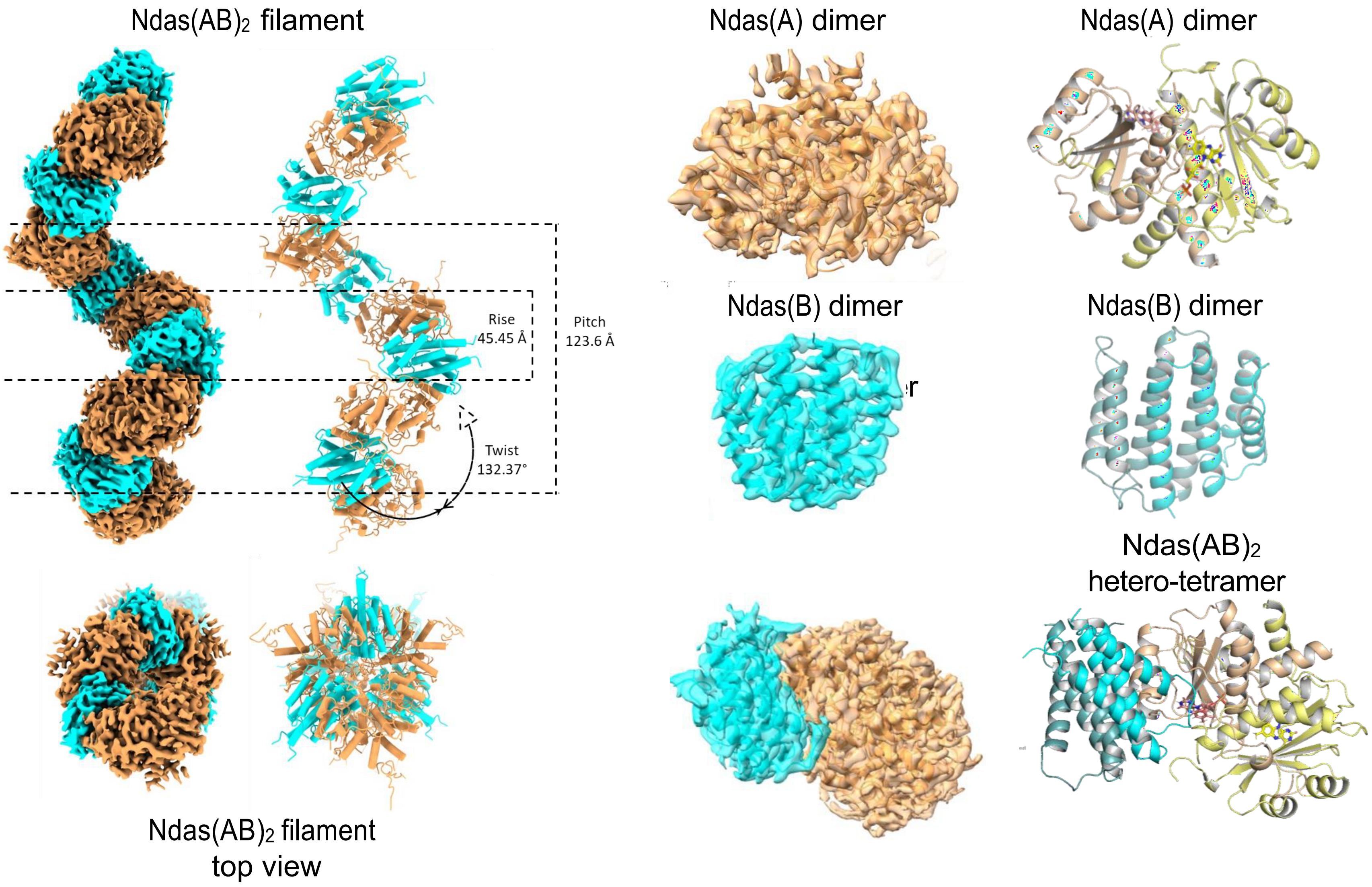

Supplementary Figure 5: Overview of cryo-EM structure.

A subunits are in tan, B subunits in cyan. a) overview of filament; b) Maps for subunits (left) as well the cartoon representation. Cryo-EM models for subunits alone and in combination (bottom depicts the dimer of dimers).

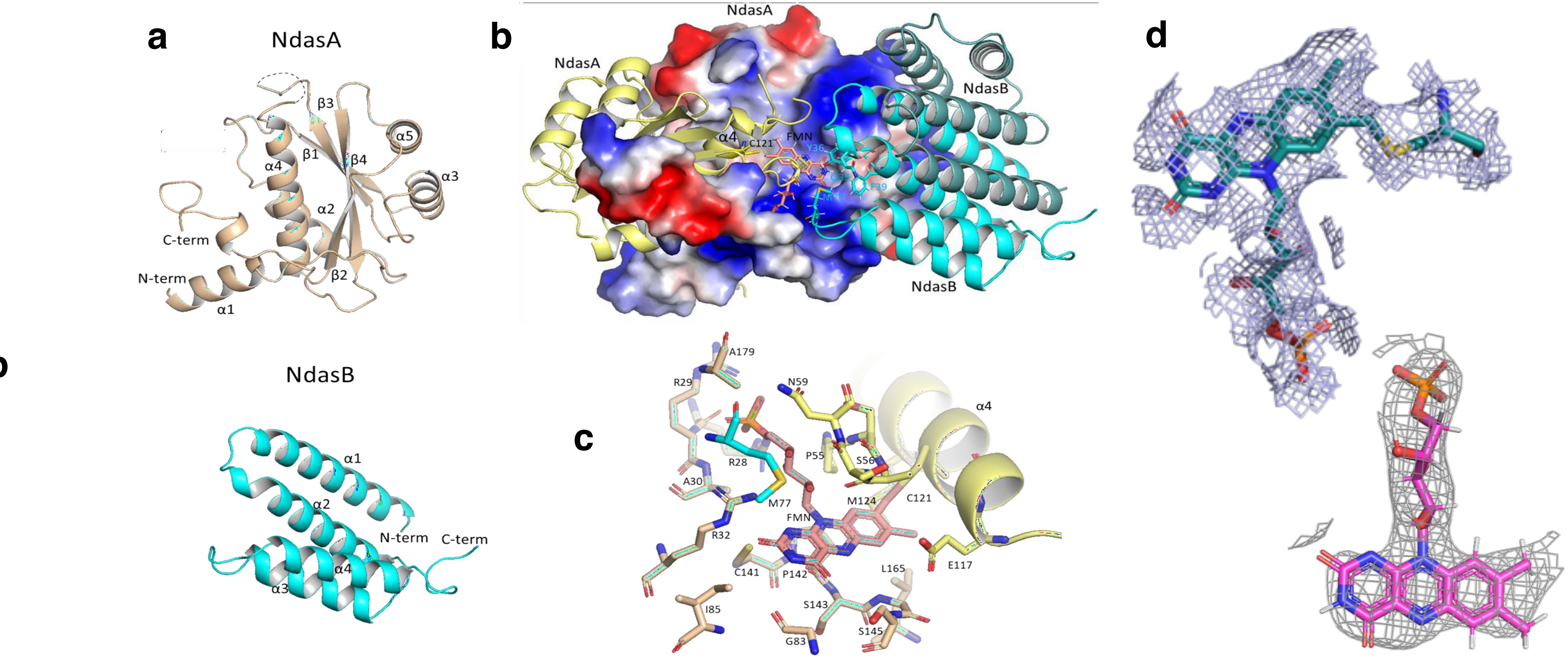

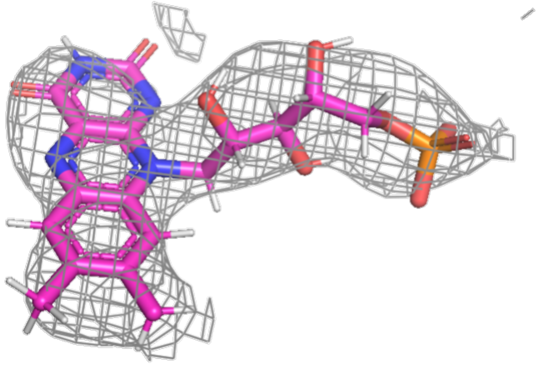

Supplementary Figure 6: Details on the FMN binding site and overall structure of A and B subunits.

a) Topology for individual A and B subunits. b) Electrostatic potential surface surrounding the FMN binding site. c) Residues in the vicinity of the FMN binding site. d) Top: density map surrounding the FMN cofactor and showing continuous density towards Cys121. Bottom: FMN cofactor in two different orientations. Maps shown at 2.0 s contour, prepared using Pymol.

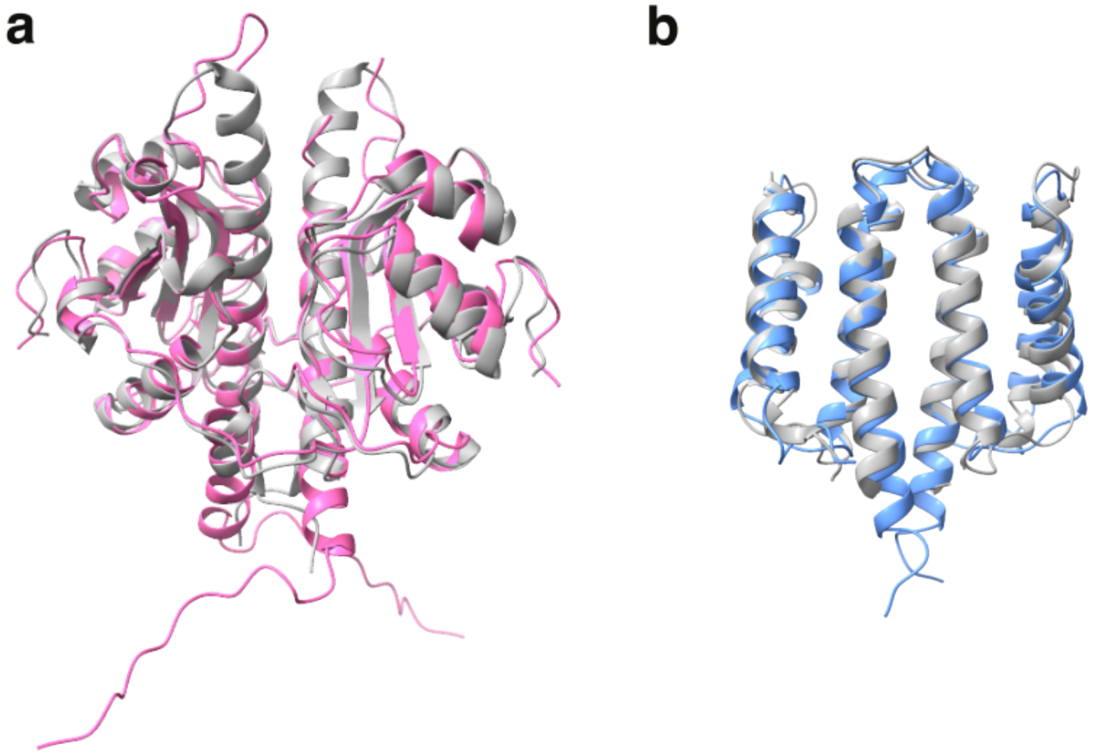

Supplementary Figure 7: Comparison of *Ndas*CDO to AlbAB[4].

AlbA and AlbB are depicted in grey whilst *Ndas*CDO-A is shown in pink and *Ndas*CDO-B in blue. Structures were compared using MatchMaker from ChimeraX, version 1.7.1. a) *Ndas*CDO-A and AlbA share an RSMD of 0.809 Å across 158 pruned atoms. b) *Ndas*CDO-B and AlbB share an RSMD of 0.877 Å across 81 pruned atoms.

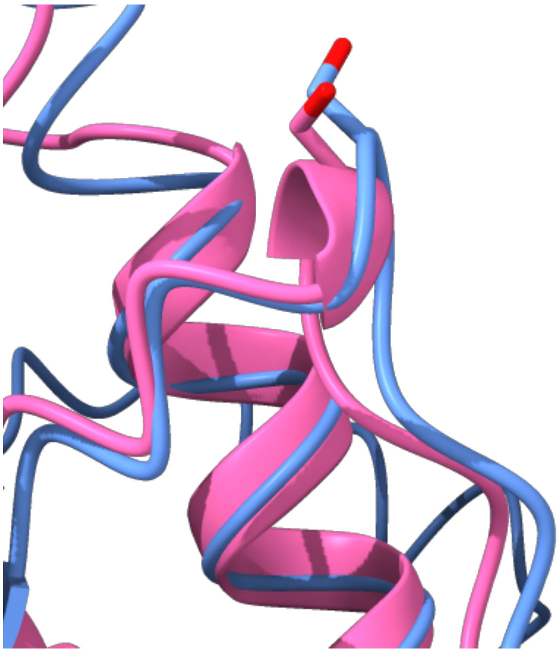

Supplementary Figure 8: Structural alignment of *Ndas*1146 with NfsA.

*Ndas*1146 structure (pink), aligned with NfsA (blue), a homolog from the nitroreductase family. Catalytic Ser41 of NfsA (PDB: 1F5V[5]) aligns with Ser58 of Ndas1146 leading to mutational analysis of this residue to investigate potential mechanistic properties.

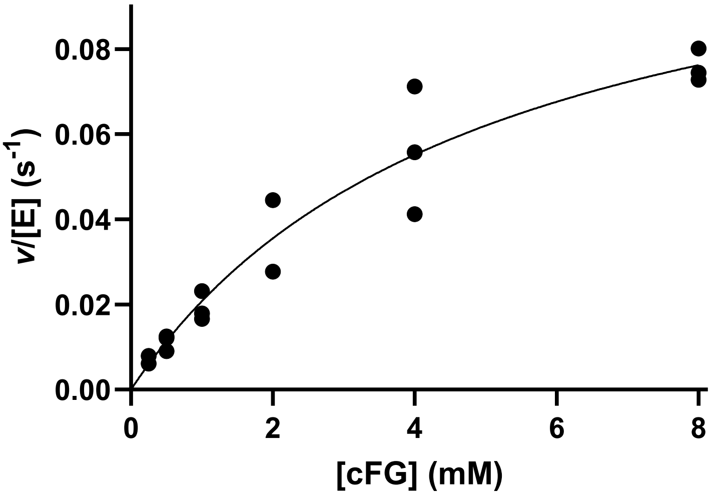

Supplementary Figure 9: NdasCDO$_{S58A}$ kinetic analysis.

Raw data for Michaelis-Menten plots using NdasCDO-S58A mutant with cFG as a substrate.

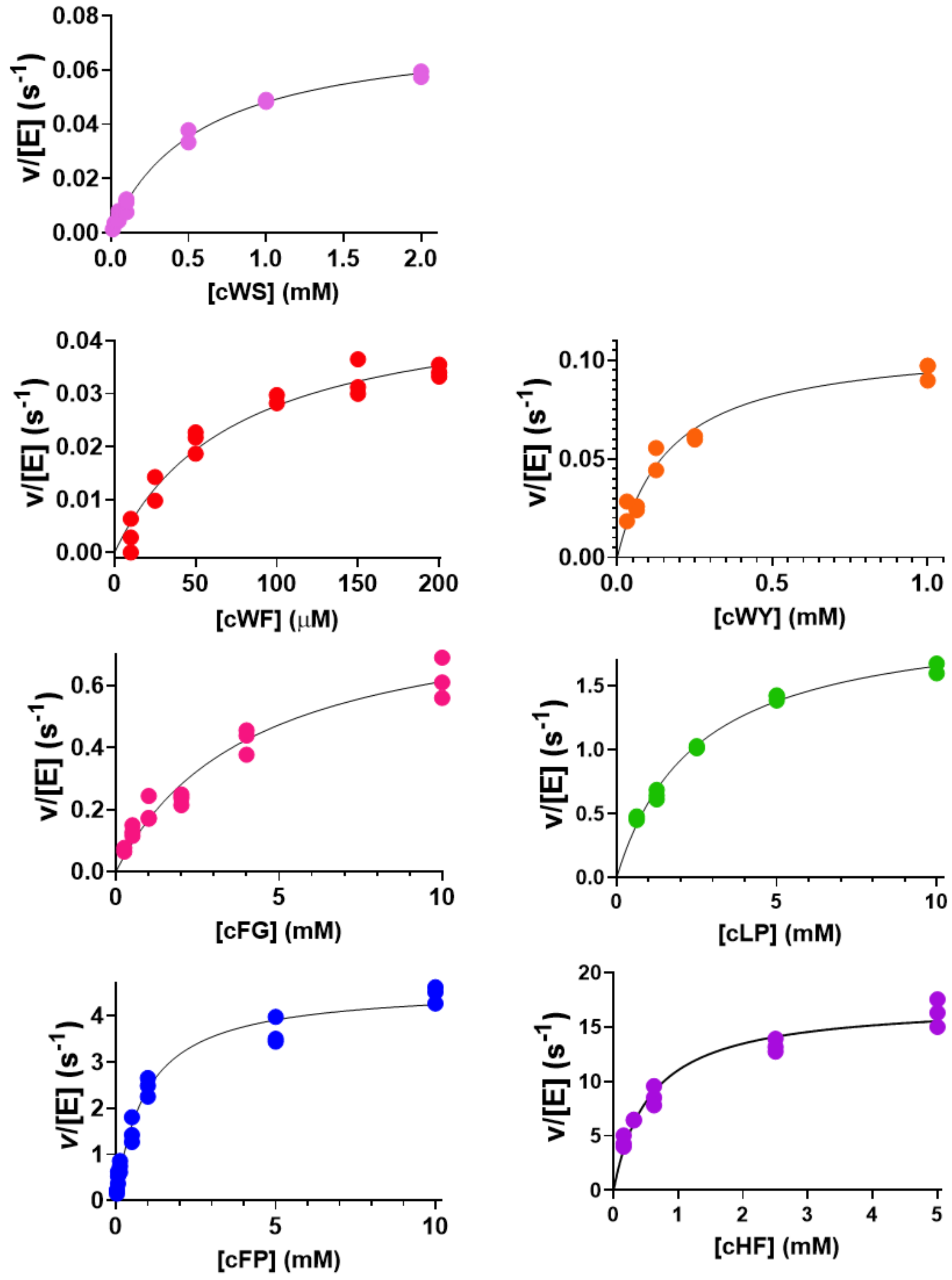

Supplementary Figure 10: Raw kinetic data related to Figure 5.

Raw data for Michaelis-Menten plots with distinct substrates. Three individual replicates per substrate concentration tested are shown.

Supplementary Figure 11: LC-MS chromatograms reporting CDP oxidation

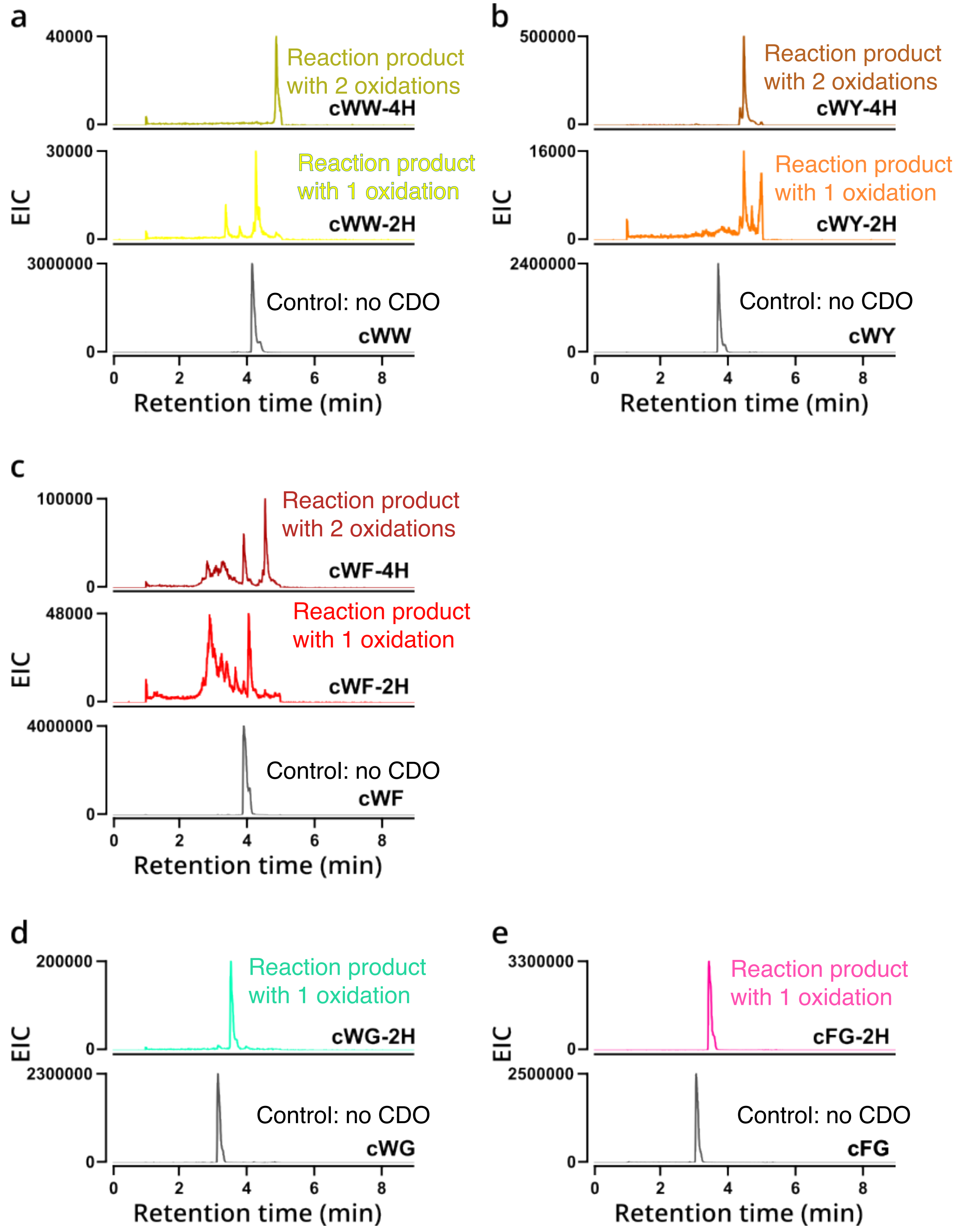

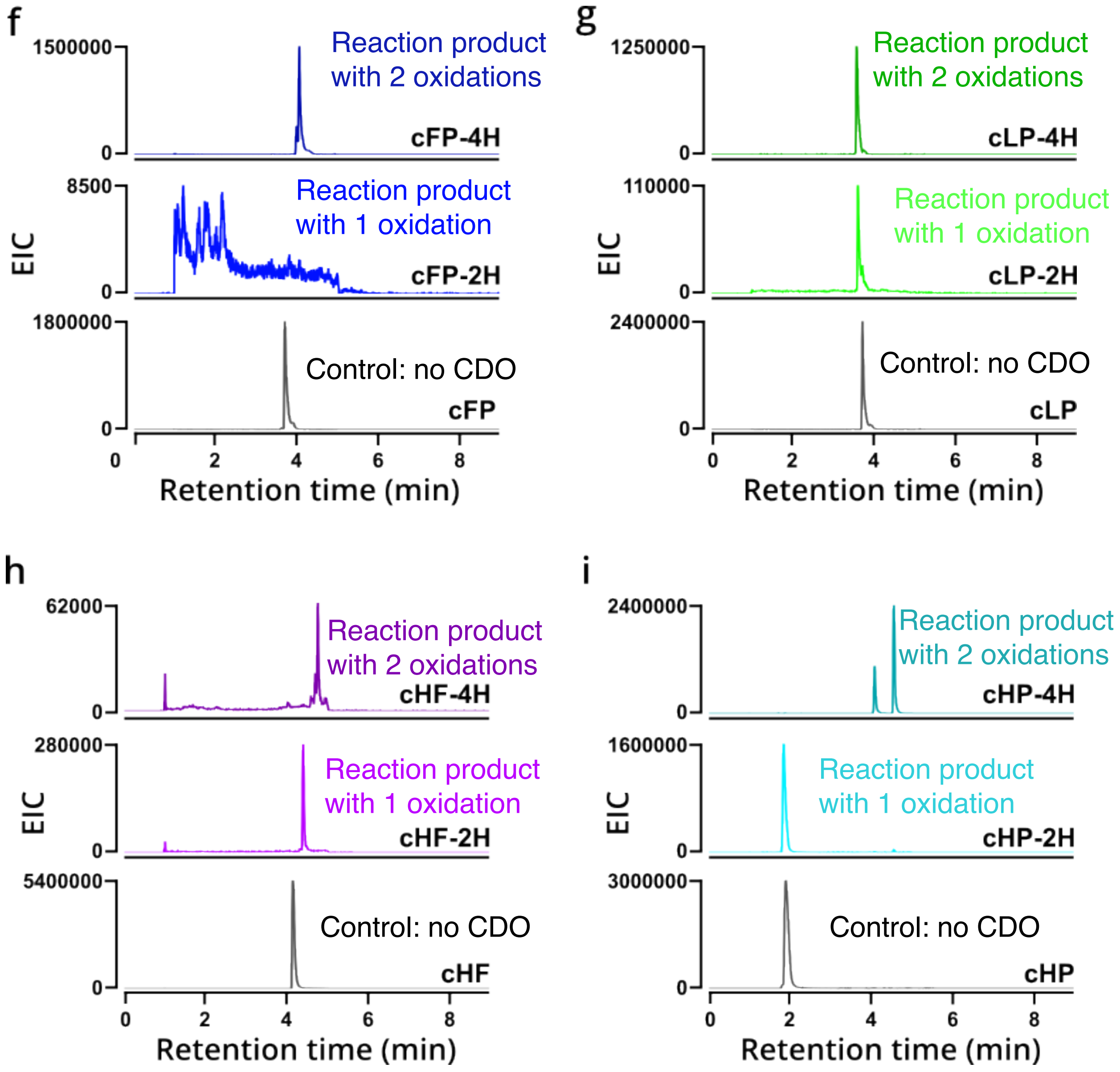

Supplementary Figure 11: LC-MS chromatograms reporting CDP oxidation. Extracted ion counts (EIC) for each CDP substrate and expected product(s) were searched within a 0.01 Da deviation and plotted separately using Prism 10.2.0. Each substrate chromatogram (in grey) was from a control experiment in absence of CDO whilst all reactions using CDO (shown in color) were incubated overnight. -2H denotes products with a single oxidation, and -4H denotes substrates that underwent two oxidation events. Panels are as follows: reaction with a) cWW, b) cWY, c) cWF, d) cWG, e) cFG, f) cFP, g) cLP, h) cHF, i) cHP.

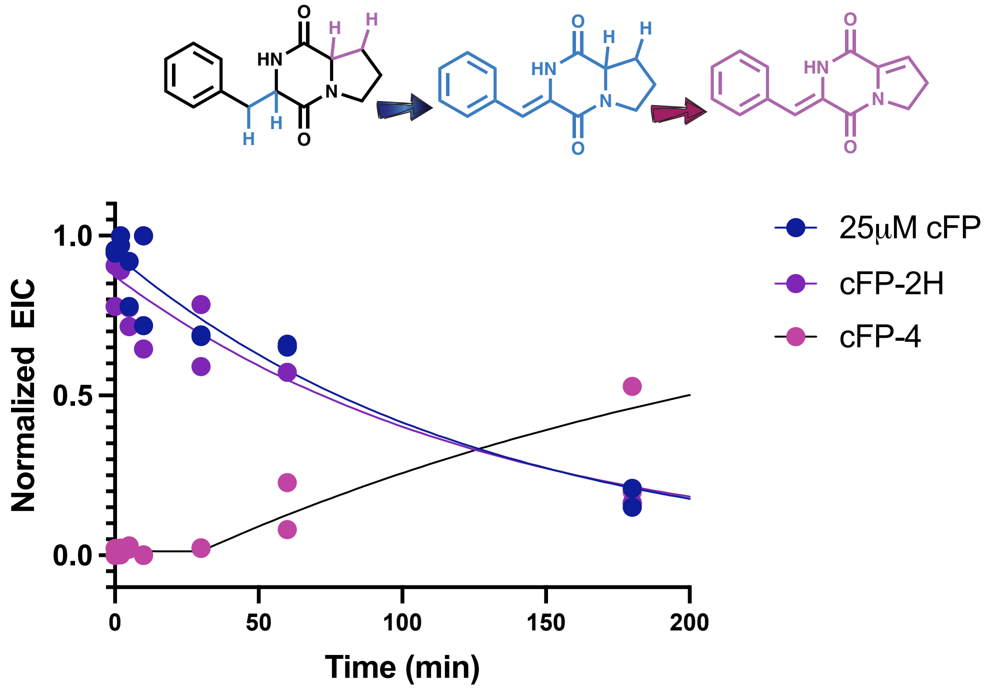

Supplementary Figure 12: Progress Curve for the reaction with cFP.

LC-MS progress curves monitoring cFP disappearance and appearance of cFP-2H and cFP-4H. Data were fitted to single exponential equations to aid data visualization. No mechanistic interpretation of rates observed is made, apart from the conclusion that there is a lag before formation of cFP-4H.

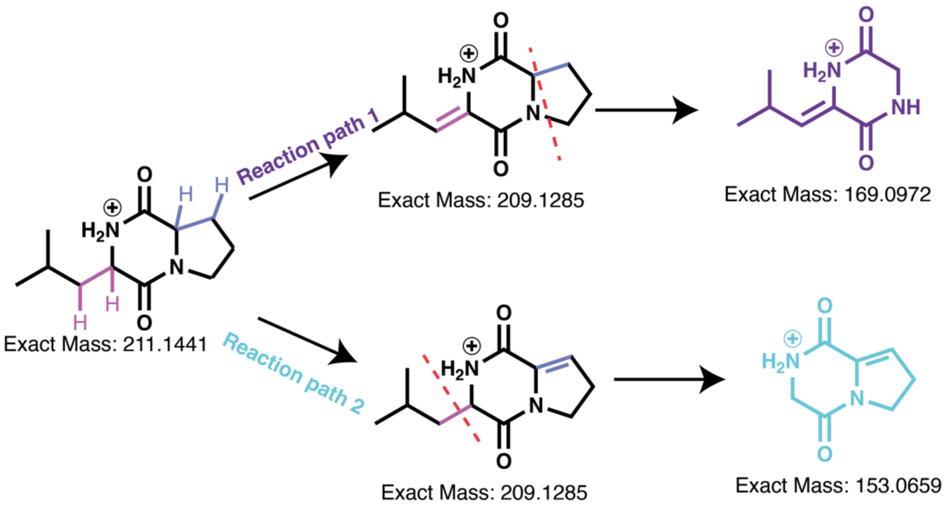

Supplementary Figure 13: Unique fragmentation products expected after oxidation of cLP.

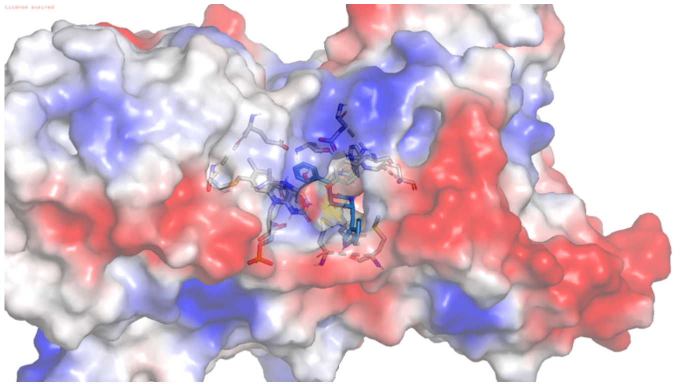

Substrate orientation	Binding Energy kcal/mol conf0	Binding Energy kcal/mol conf1	Substrate orientation
cFG	-3.4		
cFP	4.2	7.2	cPF
cFH	1.4	6.2	cHF
cPL	2.7	0.3	cLP
cFW	15.7	15.5	cWF
cWS	8.1	6.8	cSW
cYW	10.3	11.13	cWY

cFG

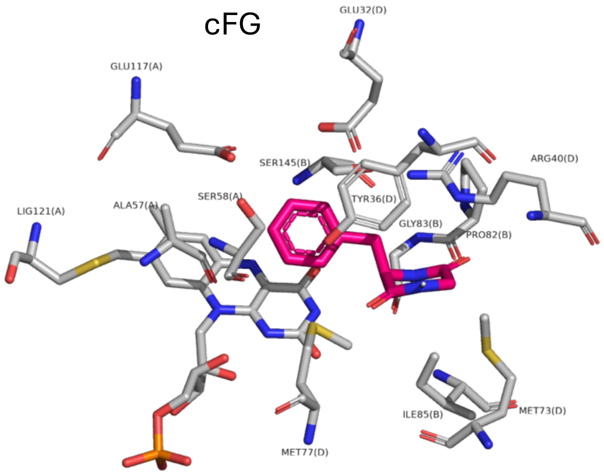

cFP_conf0

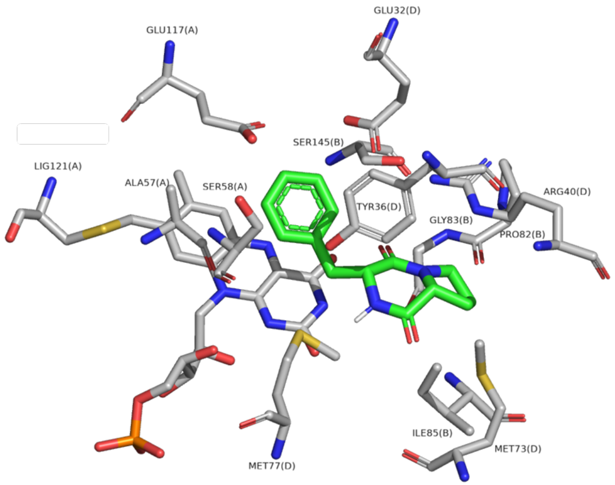

cFP_conf1

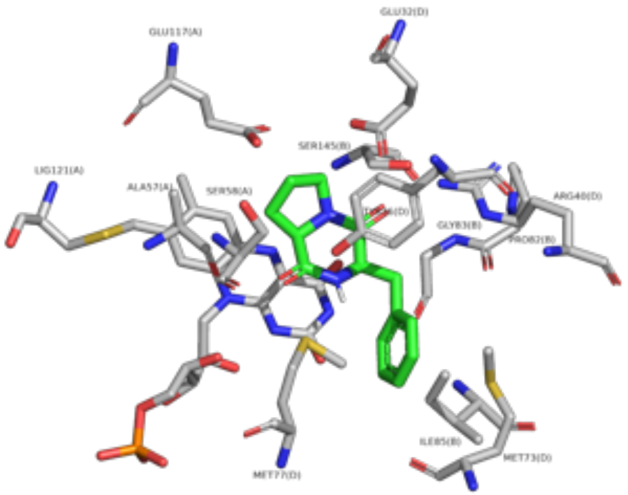

cWF_conf0

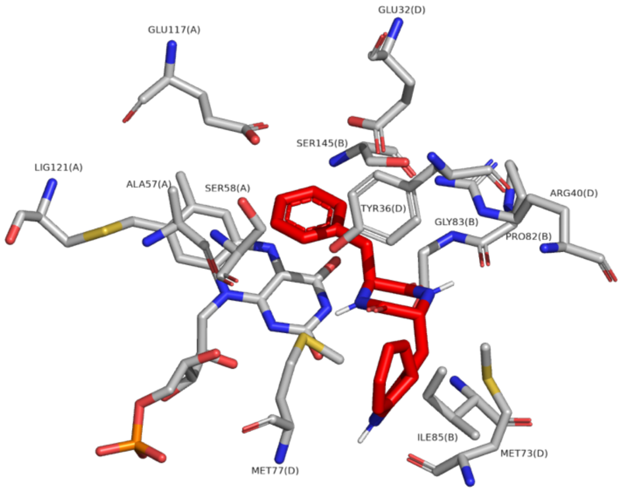

cWF_conf1

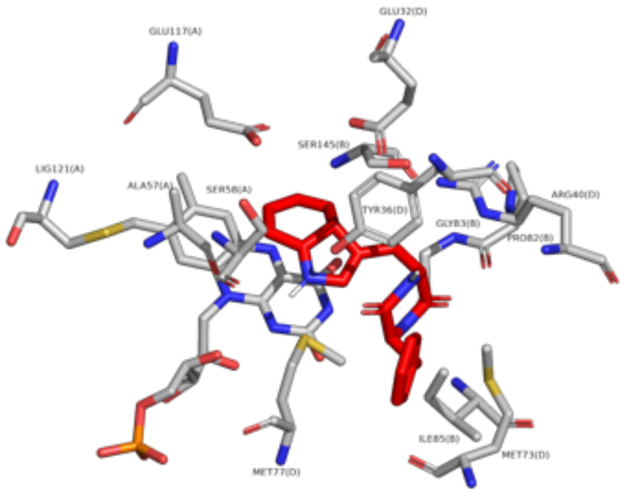

cHF_conf0

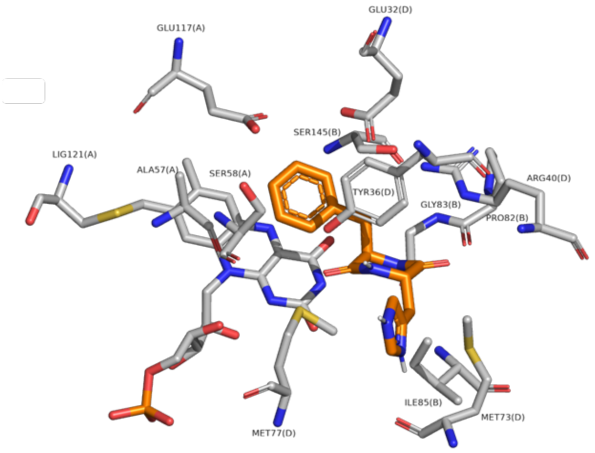

cHF_conf1

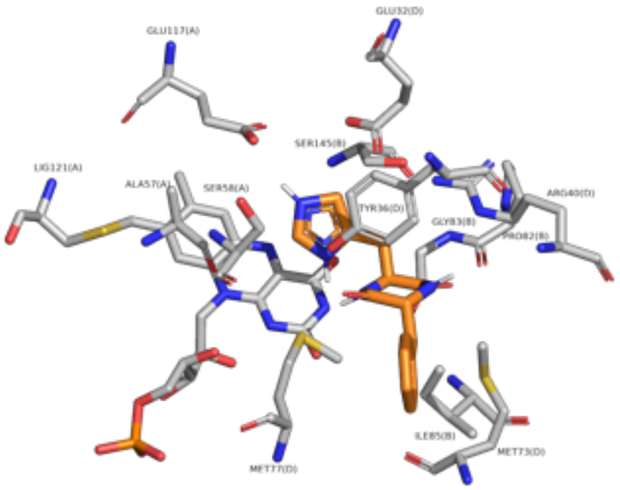

cWS_conf0

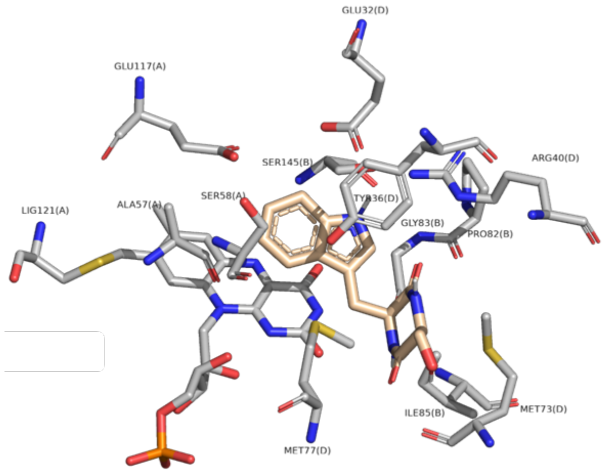

cWS_conf1

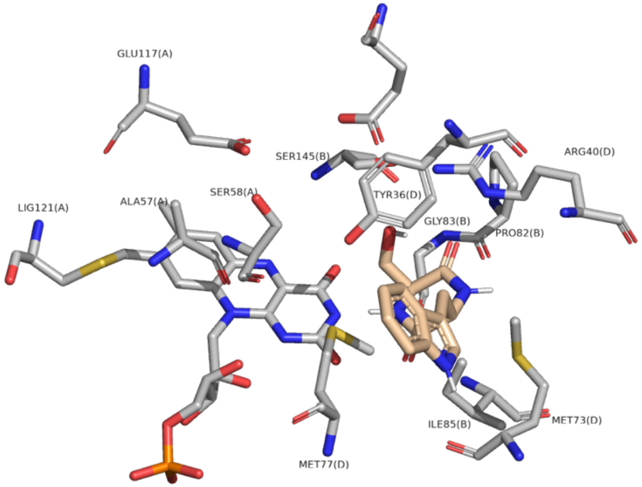

cLP_conf0

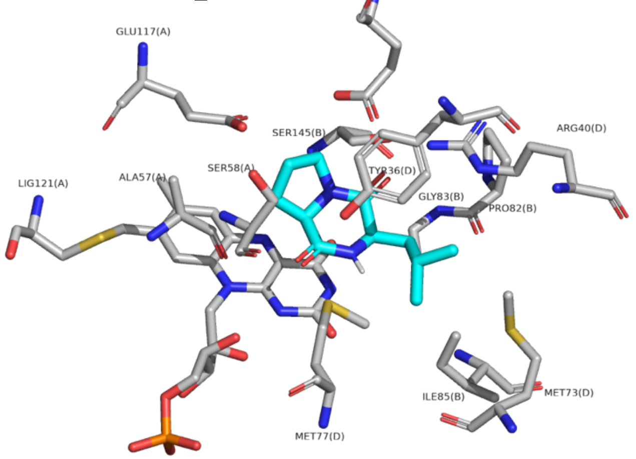

cLP_conf1

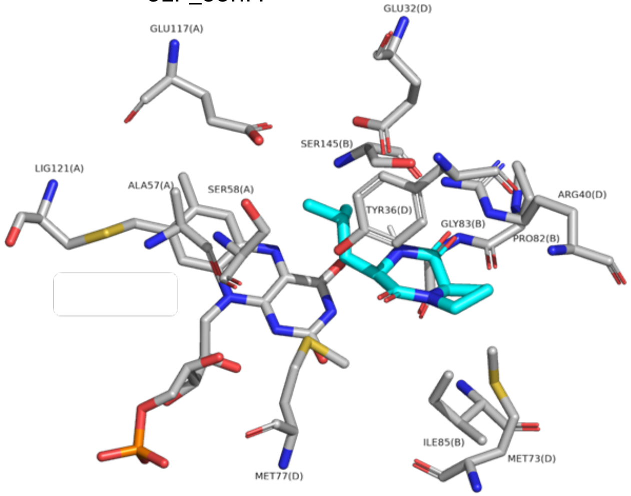

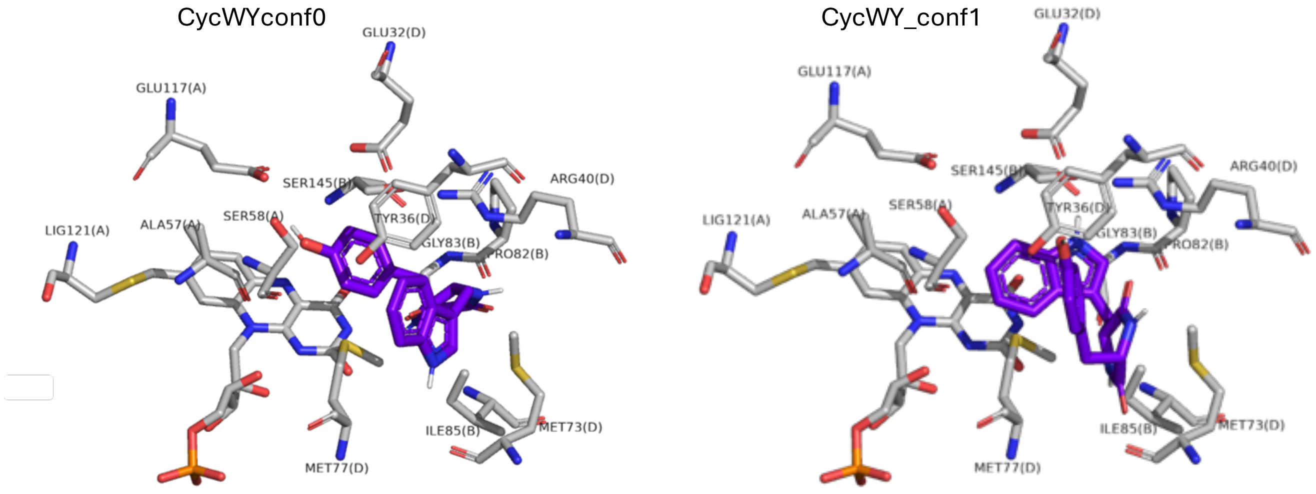

Supplementary Figure 14: Docking of CDP substrates.

Docking simulations of each CDP into the active site of *Ndas*CDO. Two possible conformations are shown where relevant and the calculated binding energy is underneath each configuration. Docking was performed using Autodock4 suite (version 4.2.6).[2] Protein is shown in grey and docked cyclic dipeptides in coloured sticks.

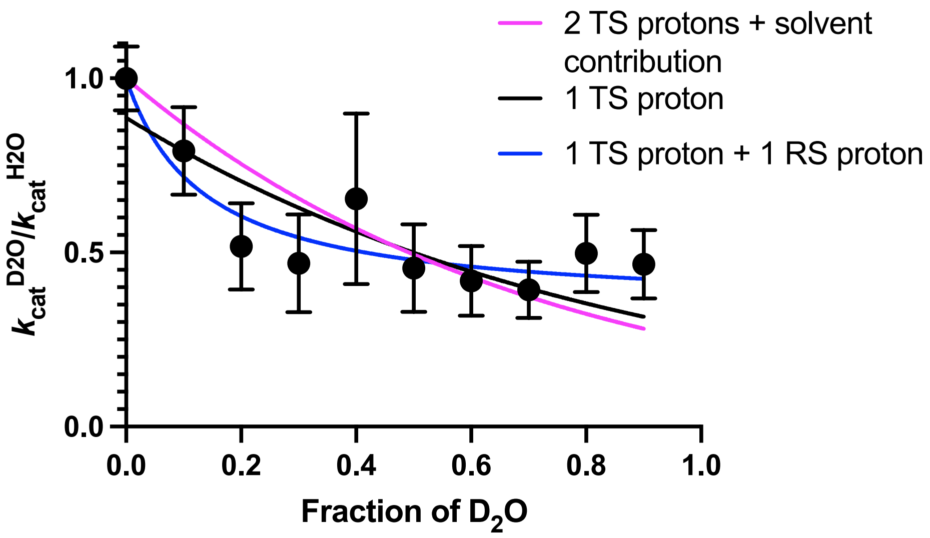

Equations for fitting:
Y= (1-x+x*ϕ1)/(1-x+x*ϕr) – 1 TS, 1 RS (blue)
Y= (1-x+x*ϕ1)^2*(ΦSolvent)^x – 2TS + bulk solvent contribution (pink)
Y= (1- x + x*ϕ1) – 1TS (black)

Supplementary Figure 15: Proton inventories for the NdasCDO-catalysed reaction.

Lines are fits to different models, as specified in the Figure legend. The best fitted model accounts for 1 transition state proton and 1 reactant state proton contributing to the observed ${}^{D2O}V$ = 2.1 ± 0.1. Average and standard error of the mean from six independent experiments.

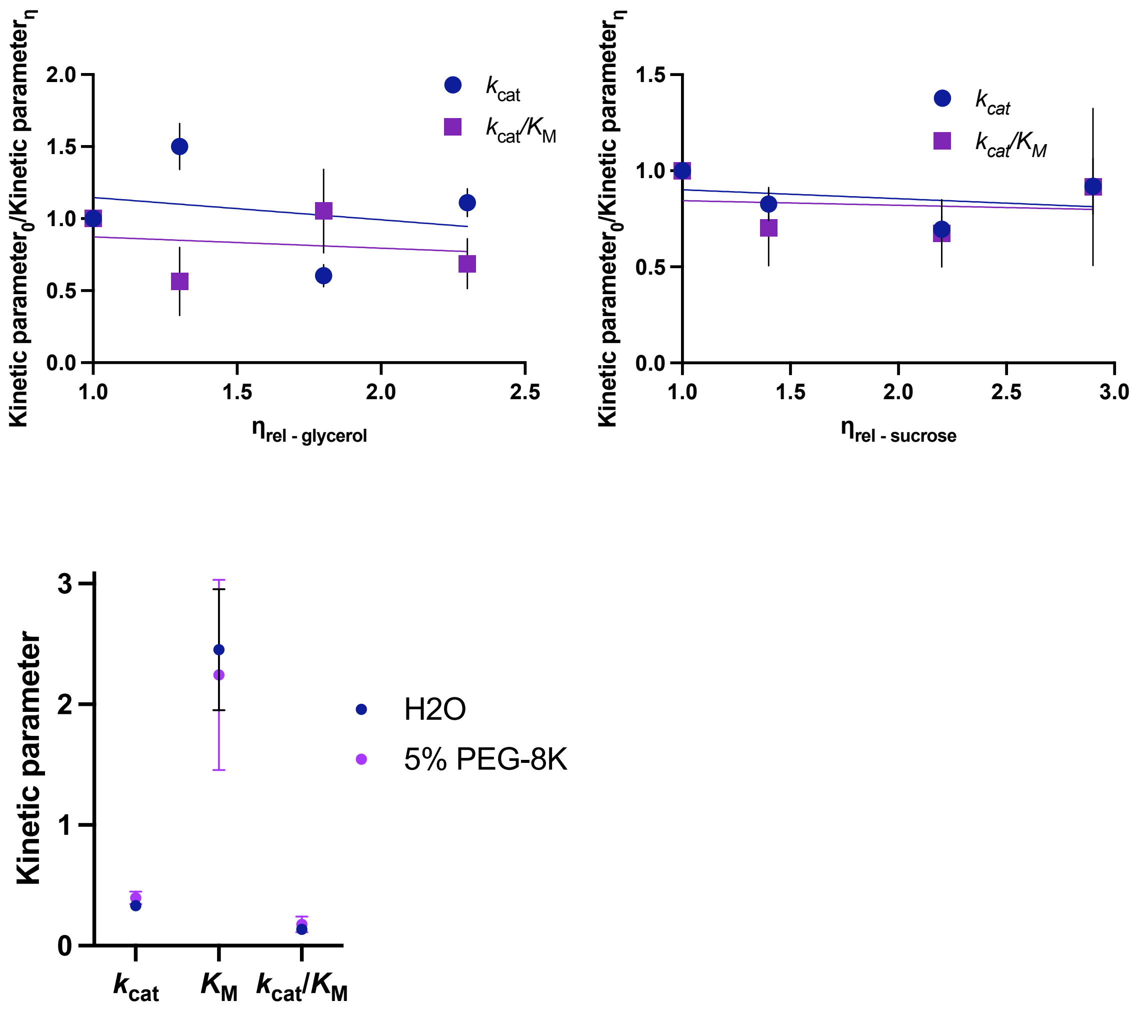

Supplementary Figure 16: NdasCDO Viscosity studies with glycerol, sucrose and PEG-8K.

Fitted kinetic parameters in the presence of increasing solvent viscosity. Data is shown as fitted value for each kinetic parameter and fitting error.

Supplementary Figure 17: NdasCDO plasmid map

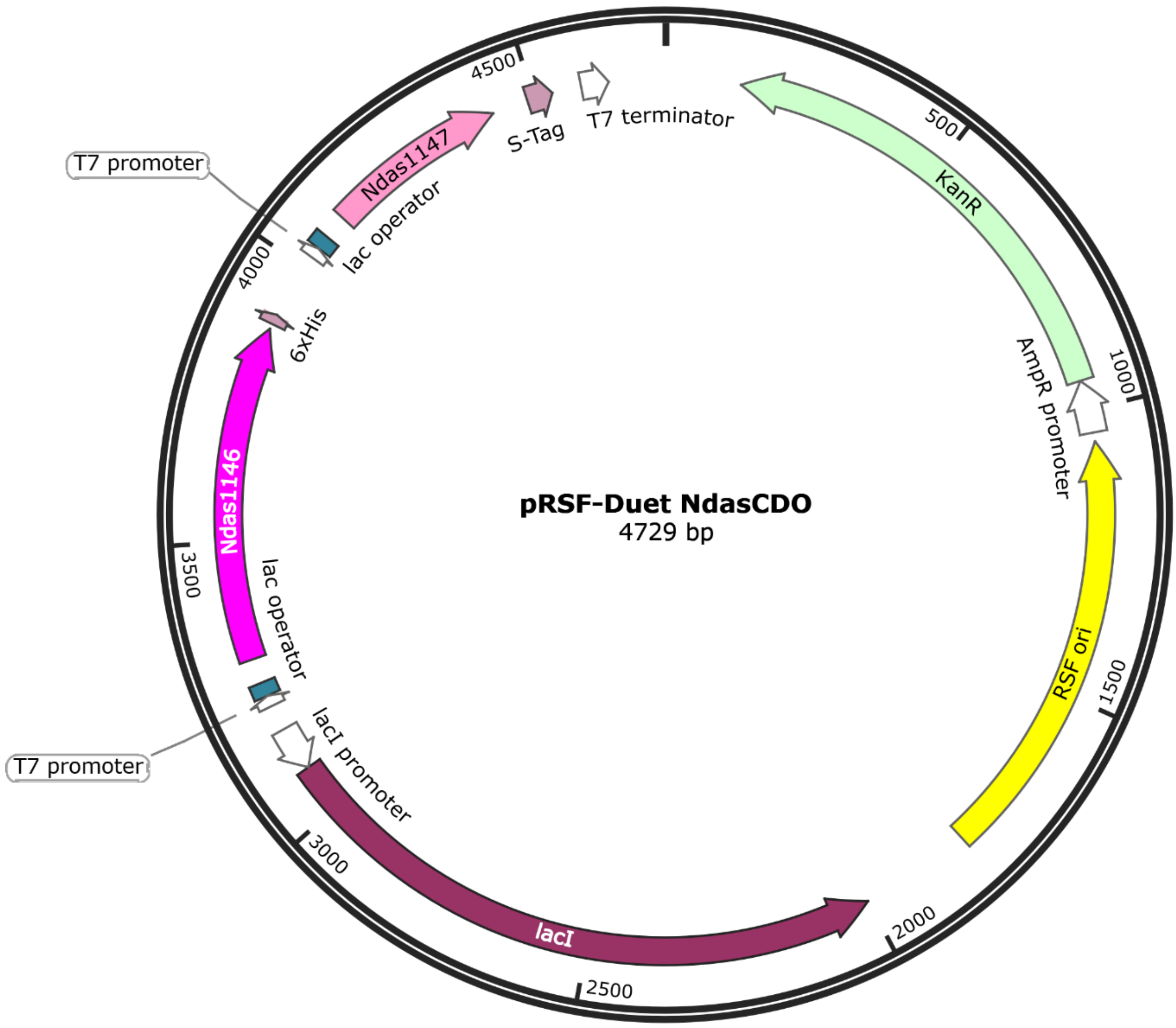

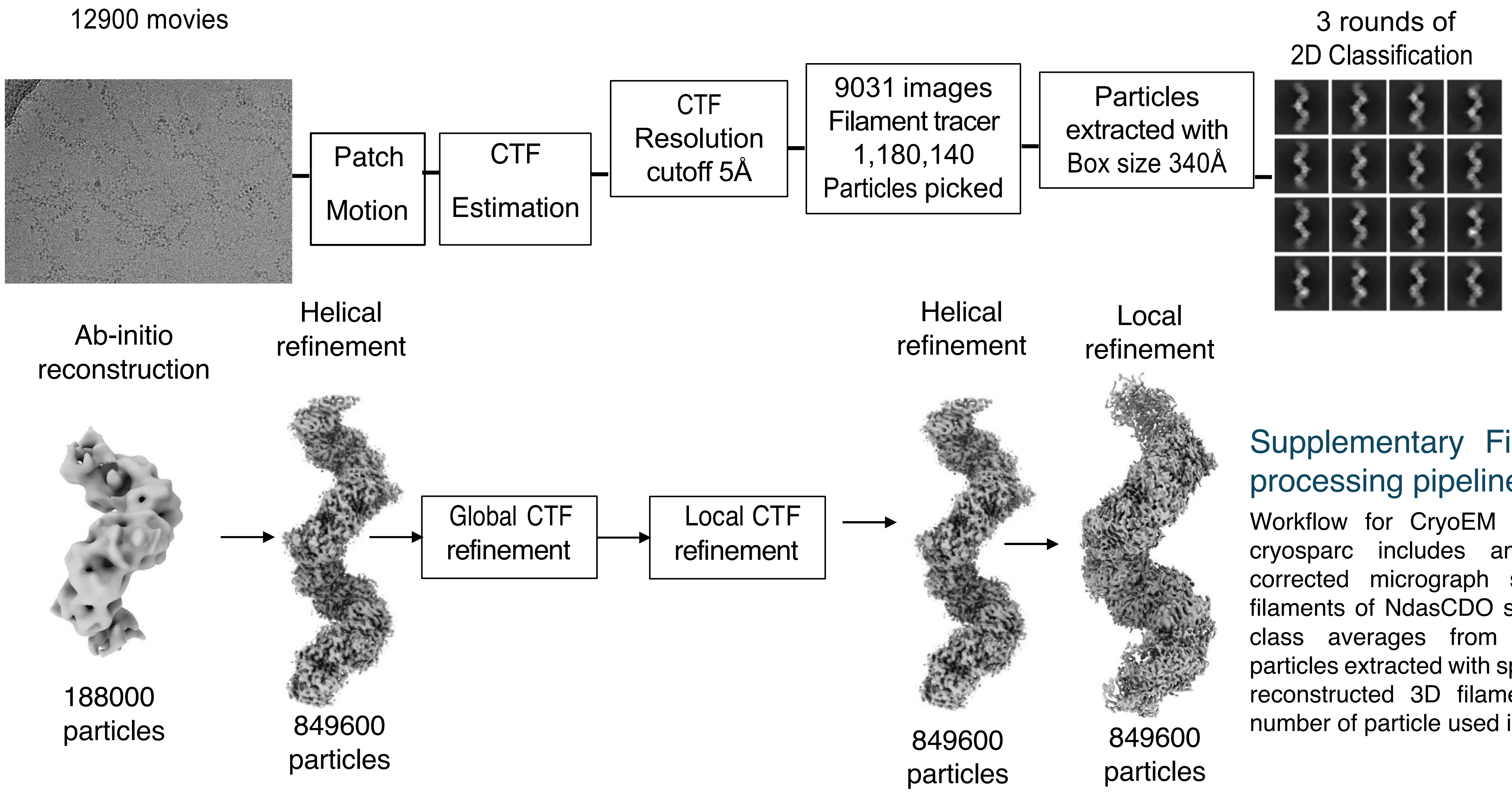

Supplementary Figure 18: Data processing pipeline.

Workflow for CryoEM data processing in cryosparc includes an example motion corrected micrograph showing distributed filaments of NdasCDO subunit A and B, 2D class averages from cleaned stack of particles extracted with specified box size and reconstructed 3D filament. At each stage number of particle used is indicated.

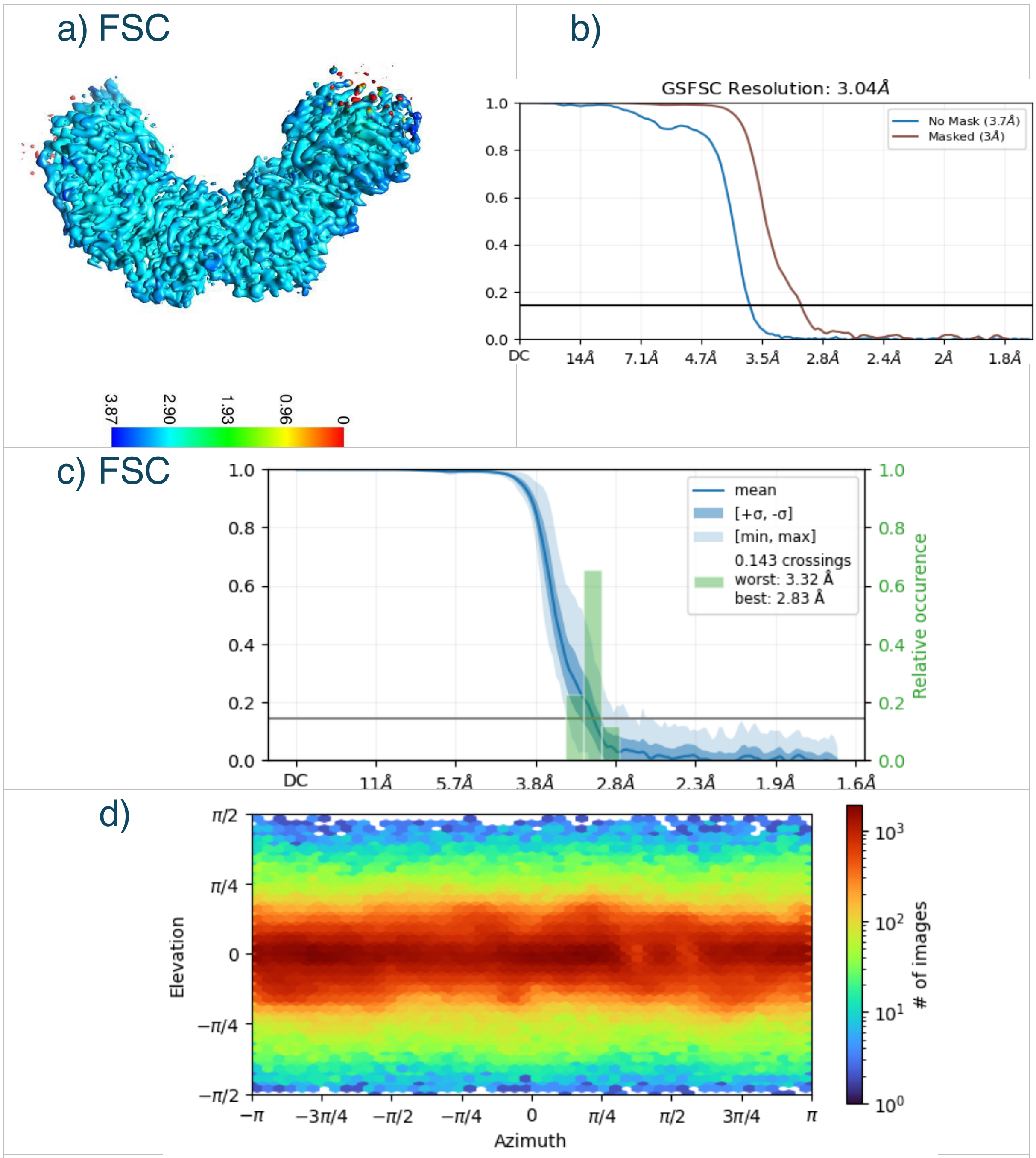

Supplementary Figure 19: Summary of reconstruction of Cryo-EM data.

a) Local resolution distribution of the reconstructed map calculated in cryosparc and color coded. b) Gold standard Fourier Shell correlation curve of masked and unmasked map. The resolution estimates at a cutoff of 0.143 crossing indicated by solid line. c) Gold standard Fourier Shell correlation curve and the estimated mean and standard deviation. At 0.143 crossing relative occurrence of worst and best resolution distribution is indicated. d) Viewing direction assigned angular value distribution.

Supplementary Tables

Supplementary Table 1: Primers for site directed mutagenesis of *Ndas*CDO

Primer	Sequence (5'-3')
2-17 truncation Fwd	TCATCACACGCCCTACAG
2-17 truncation Rev	CATGGTATATCTCCTTATTAAAGTTAAAC
2-39 truncation Fwd	GACGACTCCCTCCTCGAC
2-39 truncation Rev	CATGGTATATCTCCTTATTAAAGTTAAACAAAATTATTTC
S58A Fwd	CCCCTCGGCGGCCAACAAGCA
S58A Rev	GCGGCGAGCATGGCGTCC

Supplementary Table 2: Relative viscosities (ηrel) for different concentrations of glycerol and sucrose (measured by Bazelyansky *et al.*)

Viscogen	Concentration (%)	η_{rel}
Sucrose	14	1.5
	24	2.2
	32	2.9
Glycerol	10	1.3
	20	1.8
	30	2.3

Supplementary Table 3: Kinetic parameters for NdasCDO substrates

	k_{cat} (s^{-1})	K_M (mM)	k_{cat}/K_M (mM^{-1} s^{-1})
**cWF**	0.05 ± 0.01	0.08 ± 0.01	0.63 ± 0.21
**cWY**	0.11 ± 0.01	0.17 ± 0.02	0.65 ± 0.15
**cWS**	0.076 ± 0.002	0.57 ± 0.05	0.13 ± 0.01
**cFG**	0.87 ± 0.08	4.2 ± 0.8	0.21 ± 0.21
**cFG-S58A**	0.12 ± 0.02	4.9 ± 1.4	0.025 ± 0.0.008
**cLP**	2.07 ± 0.06	2.5 ± 0.2	0.83 ± 0.07
**cFP**	4.6 ± 0.2	0.9 ± 0.1	5.0 ± 0.1
**cHF**	17.3 ± 0.7	0.57 ± 0.08	30.6 ± 0.1

Supplementary Table 4: Data collection and refinement statistics

Data collection and processing

Magnification	105000	
Voltage (kV)	300	
Electron exposure	33.5	
Defocus range	(1.5-3.2)	
Pixel size	0.831	
Symmetry imposed	C1	
Initial particle images (no.)	1180140	
Final particle images (no.)	849600	
Map resolution (Å)	3.07	
FSC threshold	0.143	
Map resolution range (Å)	2.8-3.4	
**Helical reconstruction**		
Helical rise	45.45	
Helical twist	132.327	
Pitch	123.6	
**Refinement**		
Models	NdasA	NdasB
Initial model	Alphafold	Alphafold
Map sharpening B factor (Å^2)	180	180
Model composition		
Non-hydrogen atoms	2684	1510
Protein residues	343	189
Ligands	Cys-FMN	Nan
**r.m.s deviations**		
Bond lengths (Å)	0.003	0.08
Bond angles (°)	0.579	0.825
**Validation**		
MolProbity score	1.72	1.59
Clashscore	8.5	6.34
CaBLAM outliers (%)	2.4	1.05
Rotamer outliers (%)	0	0
Cβ outliers (%)	0	0
**Ramachandran plot**		
Favoured (%)	96.12	96.39
Allowed (%)	3.88	3.61
Disallowed (%)	0	0

Supplementary note 1: synthesis and characterization of cWS

Methodology

Thin-layer chromatography (TLC) analysis was conducted on pre-coated silica gel-coated 60 (F_{254}) and visualised with UV_{254} fluorescent indicator followed by permanganate staining solution and subsequent heating for visual enhancement.

Flash column Chromatography utilized Merck silica gel 60 (40—63 μm) range under a positive pressure from compressed air.

Nuclear magnetic resonance (NMR) spectra were obtained using two distinct setups: a Bruker Advance III 500 system featuring a Prodigy BBO cryoprobe (^{1}H, 500 MHz; ^{13}C, 125 MHz) and a Bruker Advance 400 system equipped with a BBFO probe (^{1}H, 400 MHz; ^{13}C, 125 MHz). Spectra are referenced using the stated deuterated solvent, ^{1}H NMR; DMSO-d_6 = 2.50 ppm or $CDCl_3$ = 7.26 ppm, ^{13}C NMR; DMSO-d_6 = 39.52 (± 0.06) ppm or $CDCl_3$ = 77.16 (± 0.06) ppm. The reported coupling constants (J) were provided in Hertz, accurate to the nearest decimal place. Chemical Shifts (δ) are reported in ppm (parts per million). Solvent peaks were referenced to literature values.[6]

Optical Specific Rotations were recorded with a Perkin Elmer Model 341 polarimeter at a wavelength of 589 nm (Sodium D line). The experiment utilized a cell with a 1 dm path length. Concentration (*c*) is expressed in g/100 mL using spectrophotometric grade methanol. Specific rotations determined at 20 °C.

Synthetic pathway to c(WS)

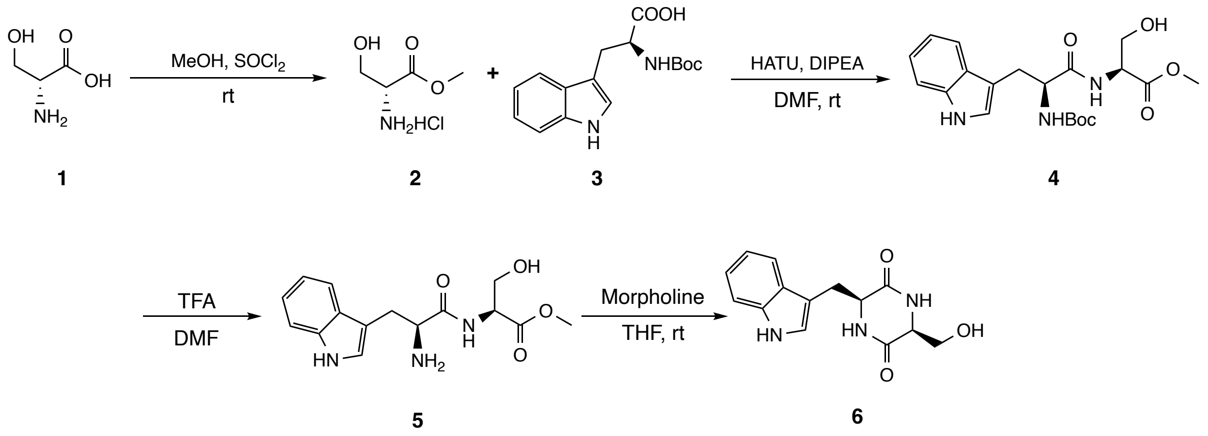

Methyl L-serinate (2)

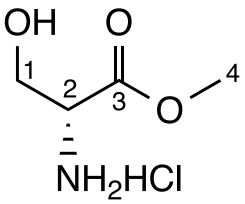

L-serine (5.00 g, 47.6 mmol) was dissolved in MeOH (64 mL) at -5 °C. Thionyl chloride (20.7 mL, 286 mmol) was slowly added over 30 minutes. The mixture was stirred at rt for 60 hours. The mixture was then concentrated followed by co-evaporation with diethyl ether four times. The resulting solid was recrystallized in MeOH to give compound **2 (4.34 g, 58%)** as white crystals. Consistent with literature data.[7]

^{1}H NMR (400 MHz, DMSO-d_6) δ 8.64 (3H, s, NH_3), 5.64 (1H, s, OH), 4.08 (1H, t, J = 3.5 Hz, H-2), 3.84-3.83 (2H, m, H-1), 3.74 (3H, s, H-4). **^{13}C NMR** (126 MHz, DMSO-d_6) δ 169.0, 59.9, 54.8, 53.2.

Boc-(L-Trp-L-Ser)-OMe (4)

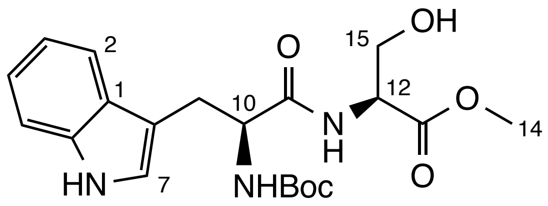

To a flame-dried flask and under nitrogen environment, Boc-tryptophan (3.00 g, 9.86 mmol), **2** (1.39 g, 8.96 mmol), HATU (3.75 g, 9.86 mmol), anhydrous DIPEA (4.7 mL, 26.88 mmol) and anhydrous DMF (20.0 mL) were added. The reaction was allowed to stir at rt until complete consumption of starting material, 16 h. The solution was washed with water, ethyl acetate (×3) and brine (×1) and dried over $MgSO_4$. Solution was then concentrated under reduced pressure, followed by co-evaporation using DCM and hexane. Purification using silica gel column chromatography (30-20% Hexanes/EtOAc) to afforded compound **4 (3.39 g, 93% yield)** as a white crystalline powder. Consistent with literature data.[8]

R_f 0.35 (20% Hexanes/EtOAc); **^{1}H NMR** (500 MHz, $CDCl_3$) δ 8.19 (1H, s, NH), 7.67 (1H, d, J = 7.9 Hz, H-2), 7.39 (1H, d, J = 8.1 Hz, H-5), 7.23 (1H, t, J = 7.5 Hz, H-4), 7.19 – 7.12 (2H, m, H-3, H-7), 6.70 (1H, d, J = 6.9 Hz, NH), 5.16 (1H, s, NH), 4.52 (1H, dt, J = 7.1, 3.4 Hz, C-12), 4.43 (1H, q, J = 6.7 Hz, C-10), 3.91-3.80 (2H, m, C-15),

3.72 (3H, s, C-14), 3.40 (1H, dd, J = 14.6, 5.9 Hz, C-9), 3.22 (1H, dd, J = 14.6, 6.7 Hz, C-9), 1.45 (9H, s, Boc); **^{13}C NMR** (126 MHz, $CDCl_3$) δ 170.3, 136.3, 127.4, 123.2, 122.5, 119.8, 118.8, 111.3, 110.4, 62.9, 55.4, 55.1, 52.7, 28.3, 27.8.

Methyl *D*-tryptophyl-*L*-serinate (5)

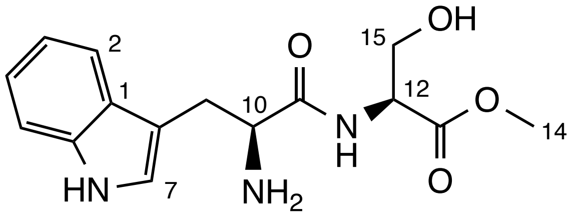

Compound **4** (3.29 g, 8.11 mmol) was added in DCM (7.0 mL) and stirred at rt until fully dissolved. TFA (3.1 mL, 40.55 mmol) was slowly added. The reaction was stirred for 16 hrs and the resulting brown solution was concentrated under reduced pressure to afford compound **5** (4.98 g) a brown viscous oil. The resulting compound was used without further purification.

R_f 0.21 (20% Hexane/Ethyl acetate); **^{1}H NMR** (500 MHz, DMSO-d_6) δ 11.05 (1H, d, J = 2.5 Hz, NH), 9.09 (1H, d, J = 7.7 Hz, NH), 8.10 – 8.06 (2H, m, NH_2), 7.76 (1H, d, J = 7.9 Hz, H-2), 7.38 (1H, d, J = 8.2 Hz, H-5), 7.24 (1H, d, J = 2.4 Hz, H-7), 7.11 (1H, t, J = 7.5 Hz, H-4), 7.02 (1H, t, J = 7.5 Hz, H-3), 4.47 (1H, dt, J = 7.6, 4.6 Hz, H-12), 4.14 (1H, dt, J = 9.5, 5.3 Hz, H-10), 3.81 (1H, dd, J = 11.1, 4.9 Hz, H-15), 3.69 – 3.66 (4H, m, H-15/H-14), 3.29 (1H, dd, J = 14.7, 5.4 Hz, H-9), 3.07 (1H, dd, J = 14.8, 8.7 Hz, H-9).

Cyclic(L-trp-L-ser) (6)

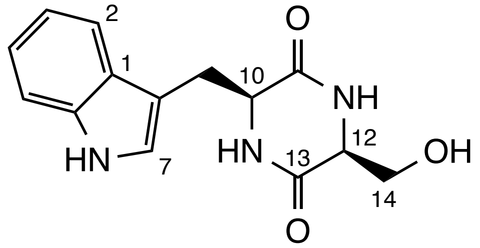

Compound **5** (2.28 g, 7.47 mmol) was dissolved in THF (21.0 ml) and stirred vigorously. The solution was brough to 0 °C. Subsequently, morpholine (9.1 ml, 104.54 mmol) was added dropwise. The solution was allowed to warm up to rt and left to stir for 48 hr. It was then concentrated under reduced pressure and purified using flash column chromatography (1-20% MeOH/DCM) to offer compound **6** (0.53 g, 26%, over 2 steps) as a white powder. Consistent with literature data.[9]

R_f 0.19 (10% methanol/DCM); $[\alpha]_D^{20}$–99.6 (*c* 0.36, MeOH); **^{1}H NMR** (500 MHz, DMSO-*d*$_6$) δ 10.88 (1H, s, NH-Indole), 7.89 (2H, m, NH x 2), 7.54 (1H, d, *J* = 7.9 Hz, H-2), 7.34 (1H, d, *J* = 8.1 Hz, H-5), 7.13 (1H, d, *J* = 2.4 Hz, H-7), 7.06 (1H, t, *J* = 7.5 Hz, H-4), 6.96 (1H, t, *J* = 7.4 Hz, H-3), 4.91 (1H, t, *J* = 5.6 Hz, OH), 4.02 (1H, dt, *J* = 6.9, 3.6 Hz, H-10), 3.68 (1H, dt, *J* = 5.7, 2.8 Hz, H-12), 3.33 – 3.28 (1H, m, H-14^a), 3.19 (2H, qd, *J* = 14.4, 5.6 Hz, H-9), 3.05 (1H, dt, *J* = 10.9, 5.6 Hz, H-14^b).**^{13}C NMR** (126 MHz, DMSO-*d*$_6$) δ 167.7, 166.2, 136.5, 128.1, 124.6, 121.3, 119.1, 118.8, 111.7, 109.6, 63.5, 57.8, 56.0, 30.9.

Selected 1H and 13C NMR spectra

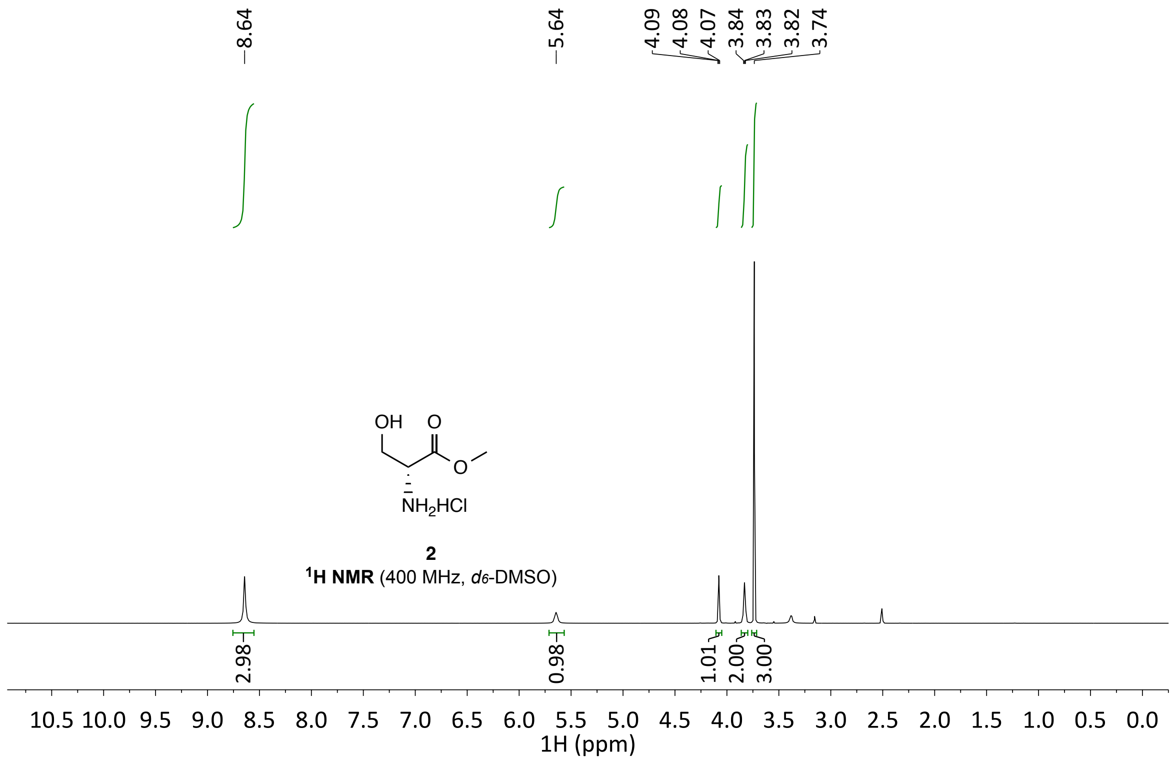

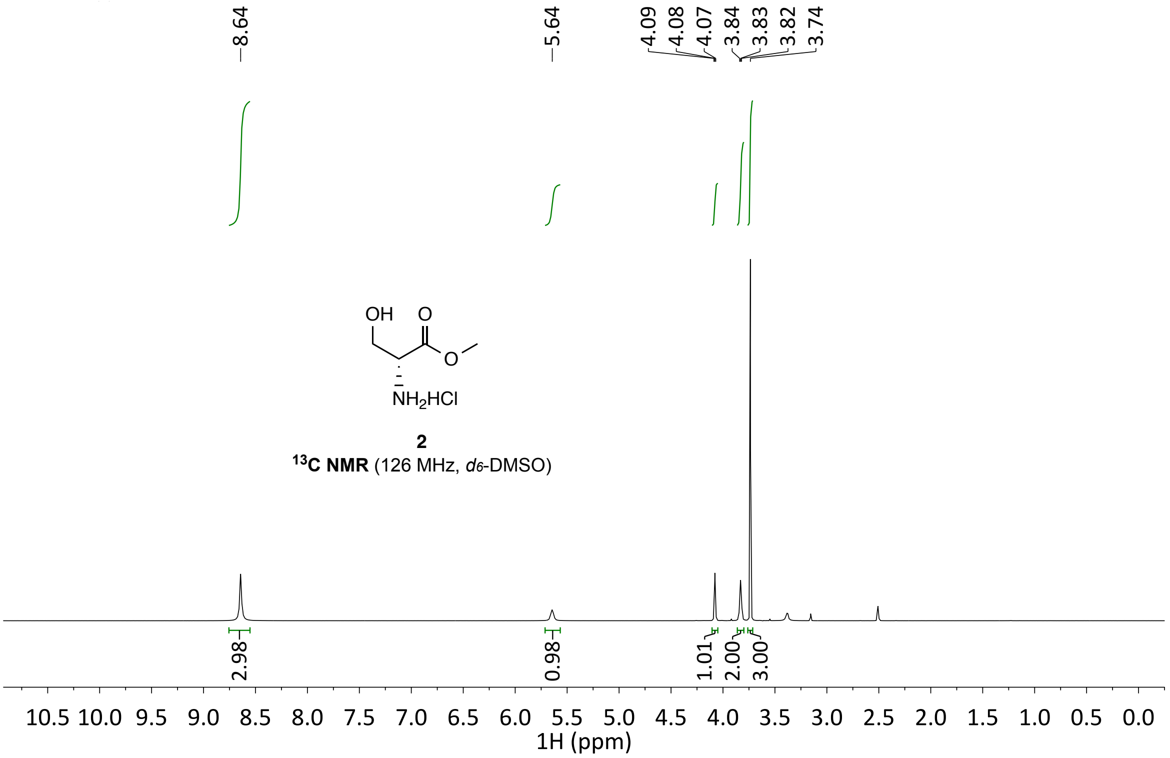
8.64
5.64
4.09 4.08 4.07 3.84 3.83 3.82 3.74
OH O
O
NH2HCl
2
13C NMR (126 MHz, d6-DMSO)
2.98 0.98 1.01 2.00 3.00
10.5 10.0 9.5 9.0 8.5 8.0 7.5 7.0 6.5 6.0 5.5 5.0 4.5 4.0 3.5 3.0 2.5 2.0 1.5 1.0 0.5 0.0
1H (ppm)

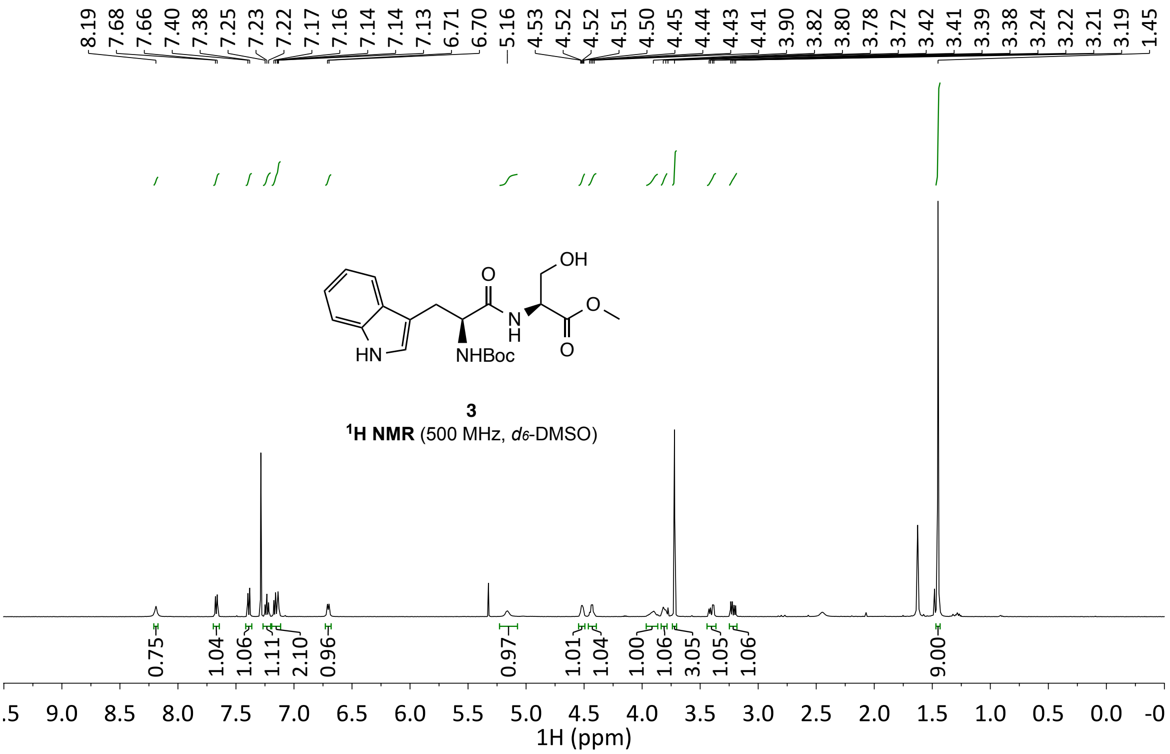
8.19 7.68 7.66 7.40 7.38 7.25 7.23 7.22 7.17 7.16 7.14 7.14 7.13 6.71 6.70
5.16
4.53 4.52 4.52 4.51 4.50 4.45 4.44 4.43 4.41 3.90 3.82 3.80 3.78 3.72 3.42 3.41 3.39 3.38 3.24 3.22 3.21 3.19 1.45
OH
O
O
N
H
HN
NHBoc
O
3
1H NMR (500 MHz, d6-DMSO)
0.75 1.04 1.06 1.11 2.10 0.96 0.97 1.01 1.04 1.00 1.06 3.05 1.05 1.06 9.00
.5 9.0 8.5 8.0 7.5 7.0 6.5 6.0 5.5 5.0 4.5 4.0 3.5 3.0 2.5 2.0 1.5 1.0 0.5 0.0 -0
1H (ppm)

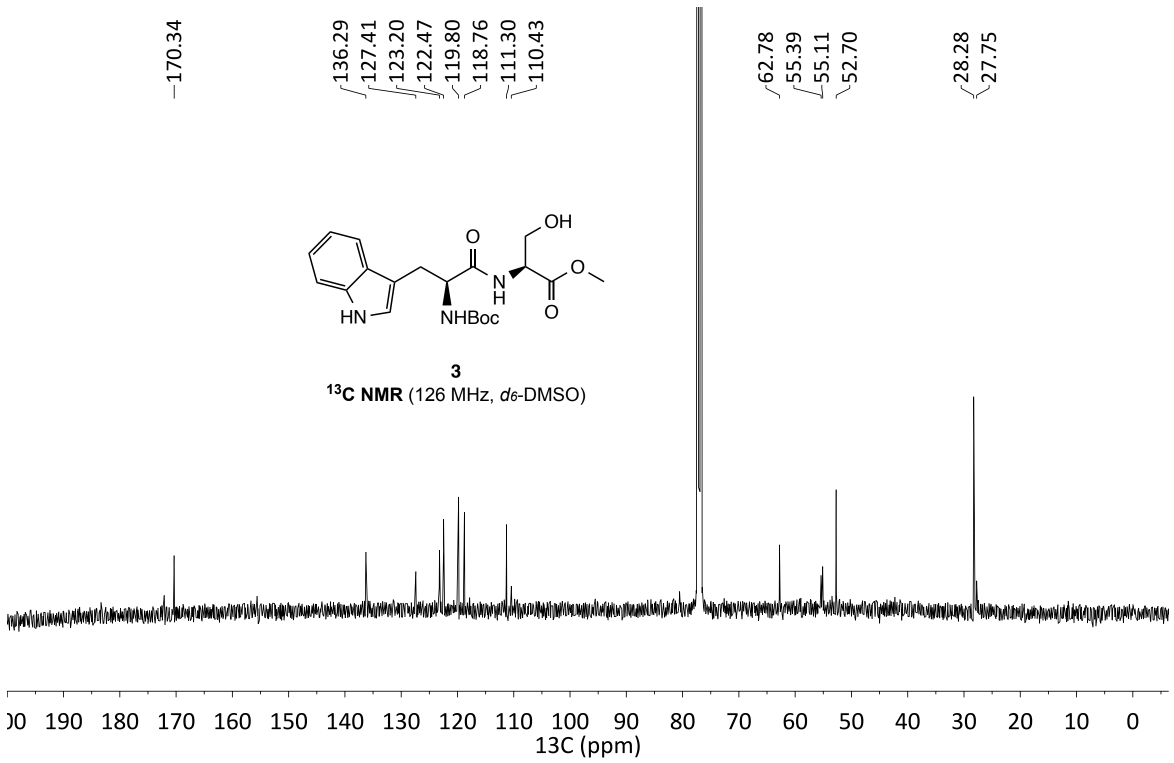

3

^{13}C NMR (126 MHz, *d6*-DMSO)

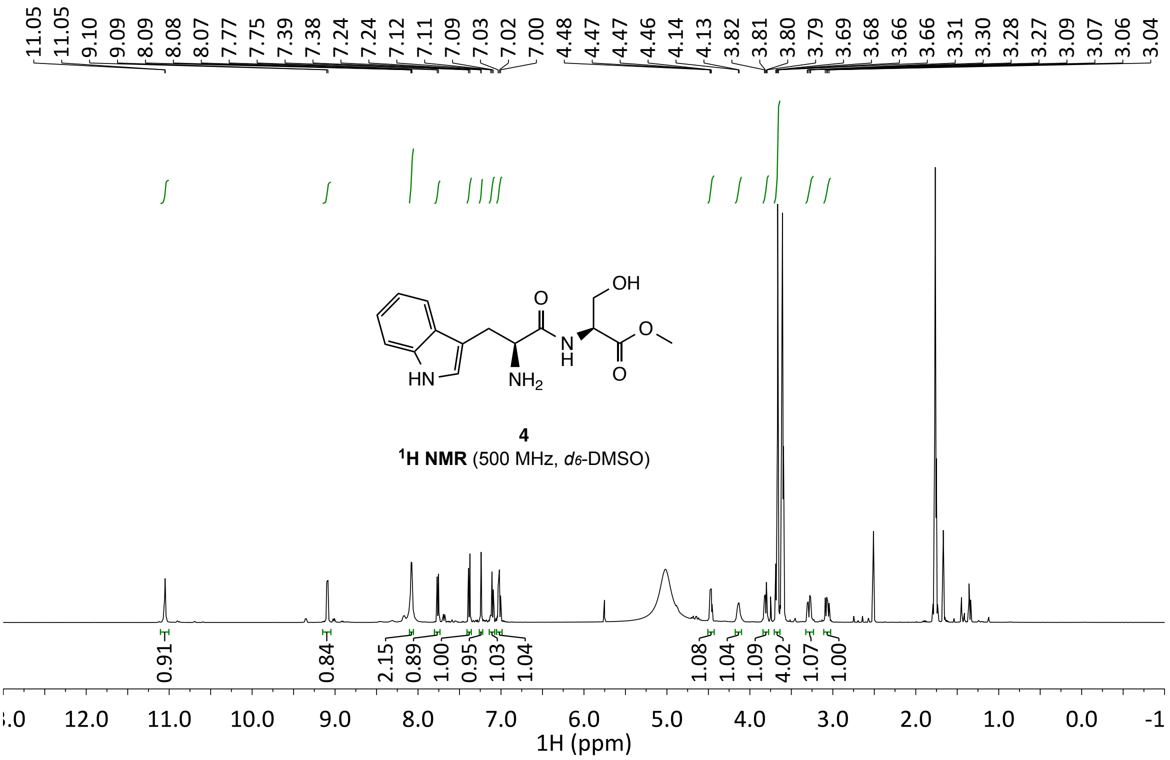
4
1H NMR (500 MHz, d6-DMSO)
1H (ppm)

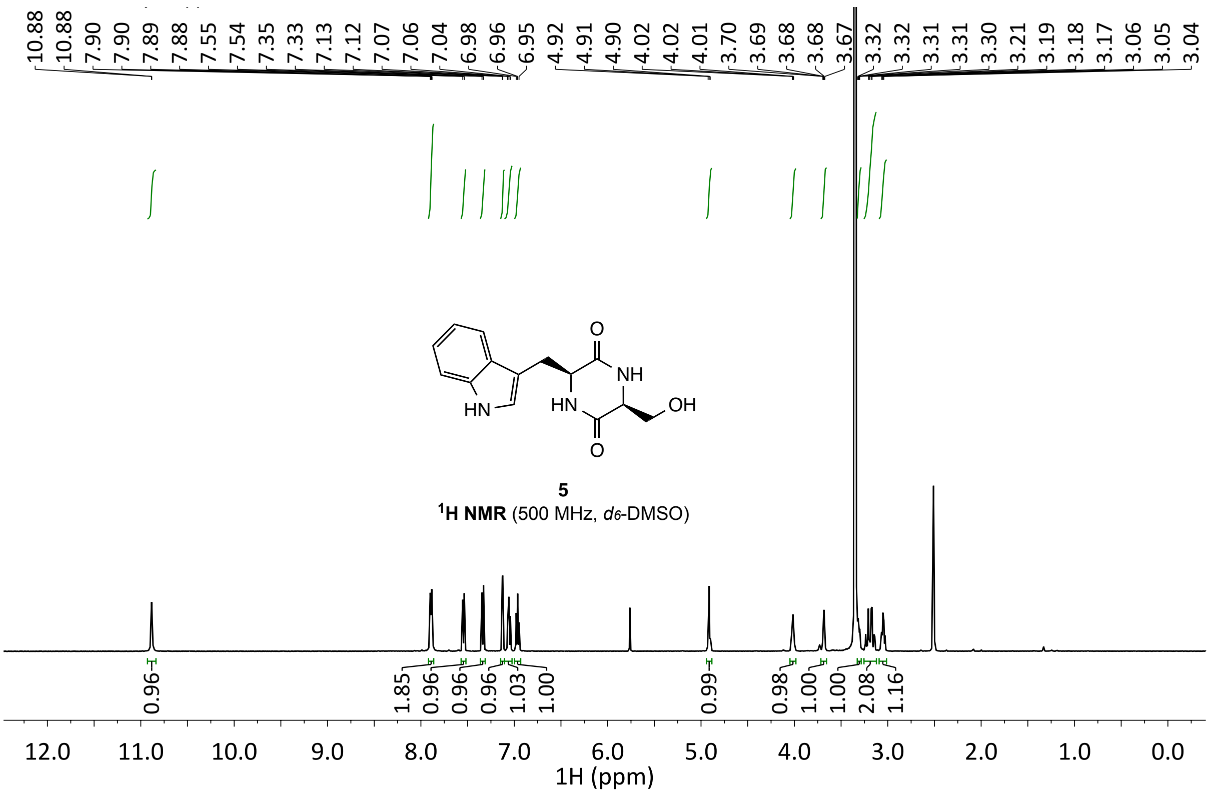
5
1H NMR (500 MHz, d6-DMSO)
1H (ppm)

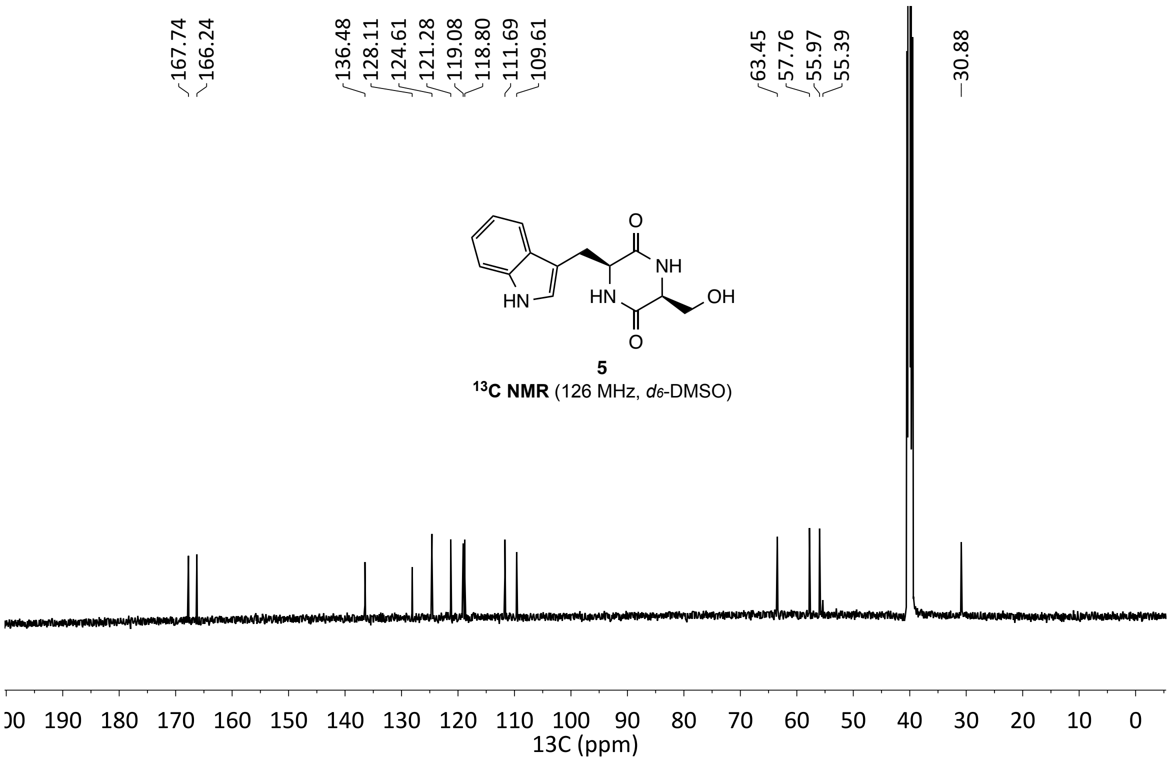
167.74
166.24
136.48
128.11
124.61
121.28
119.08
118.80
111.69
109.61
63.45
57.76
55.97
55.39
30.88
O
NH
HN
HN
OH
O
5
13C NMR (126 MHz, d6-DMSO)
13C (ppm)

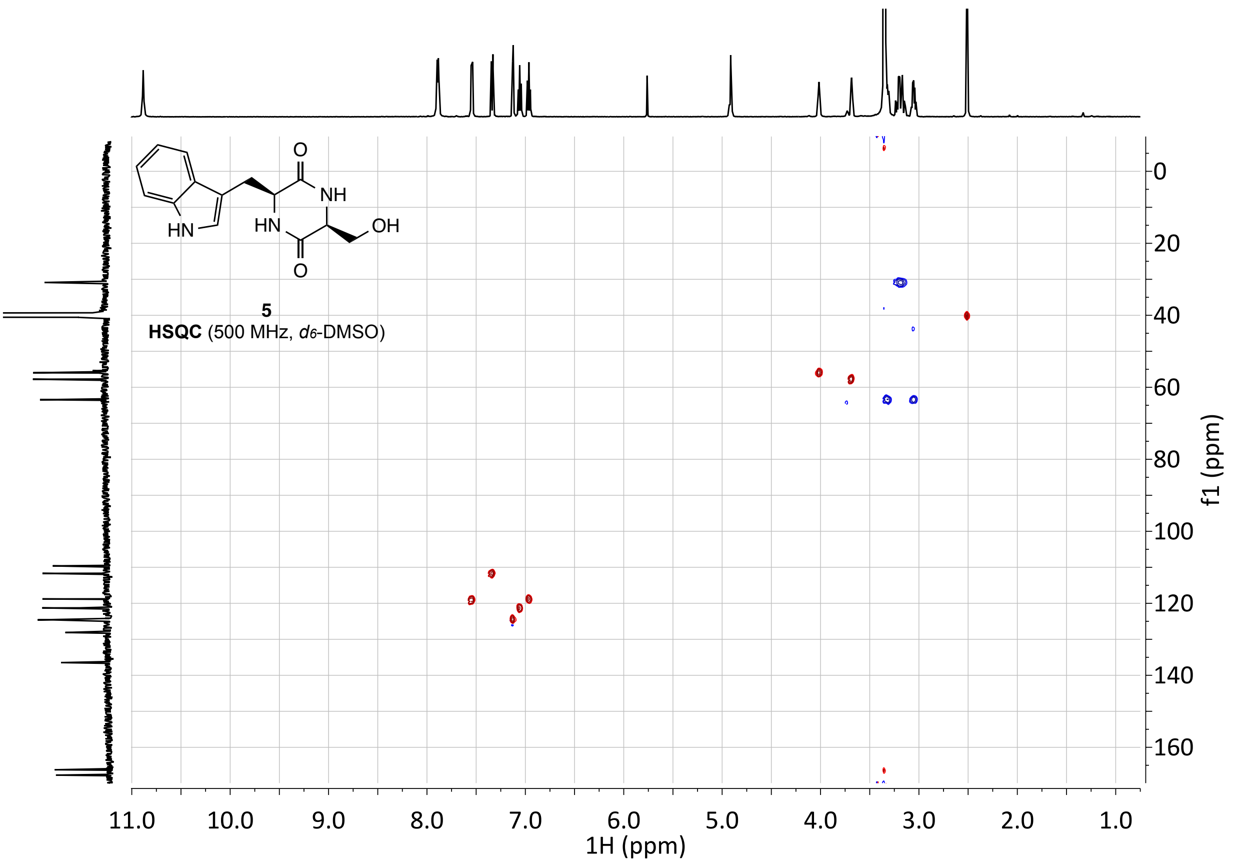
O
NH
HN
HN
OH
O
5
HSQC (500 MHz, d6-DMSO)
1H (ppm)
f1 (ppm)

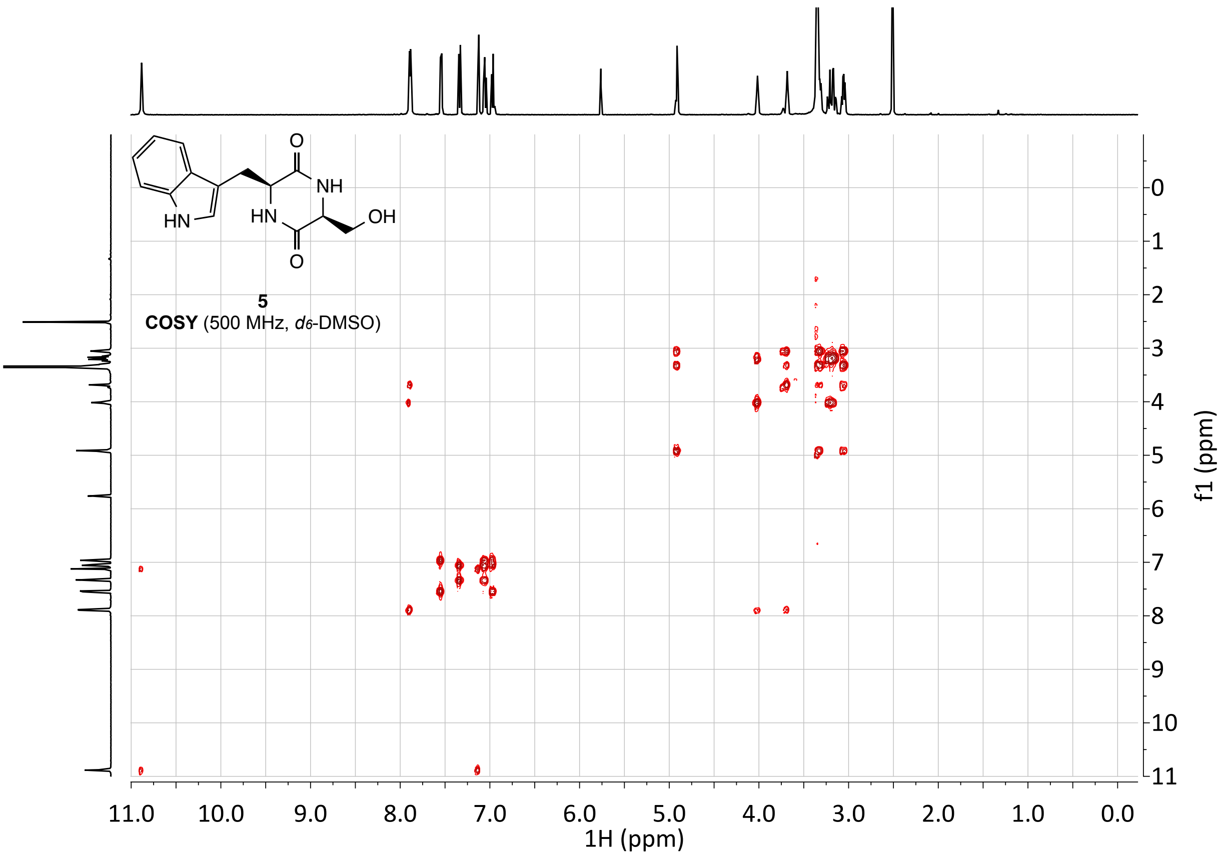
O
NH
HN
HN
OH
O
5
COSY (500 MHz, d6-DMSO)
1H (ppm)
f1 (ppm)

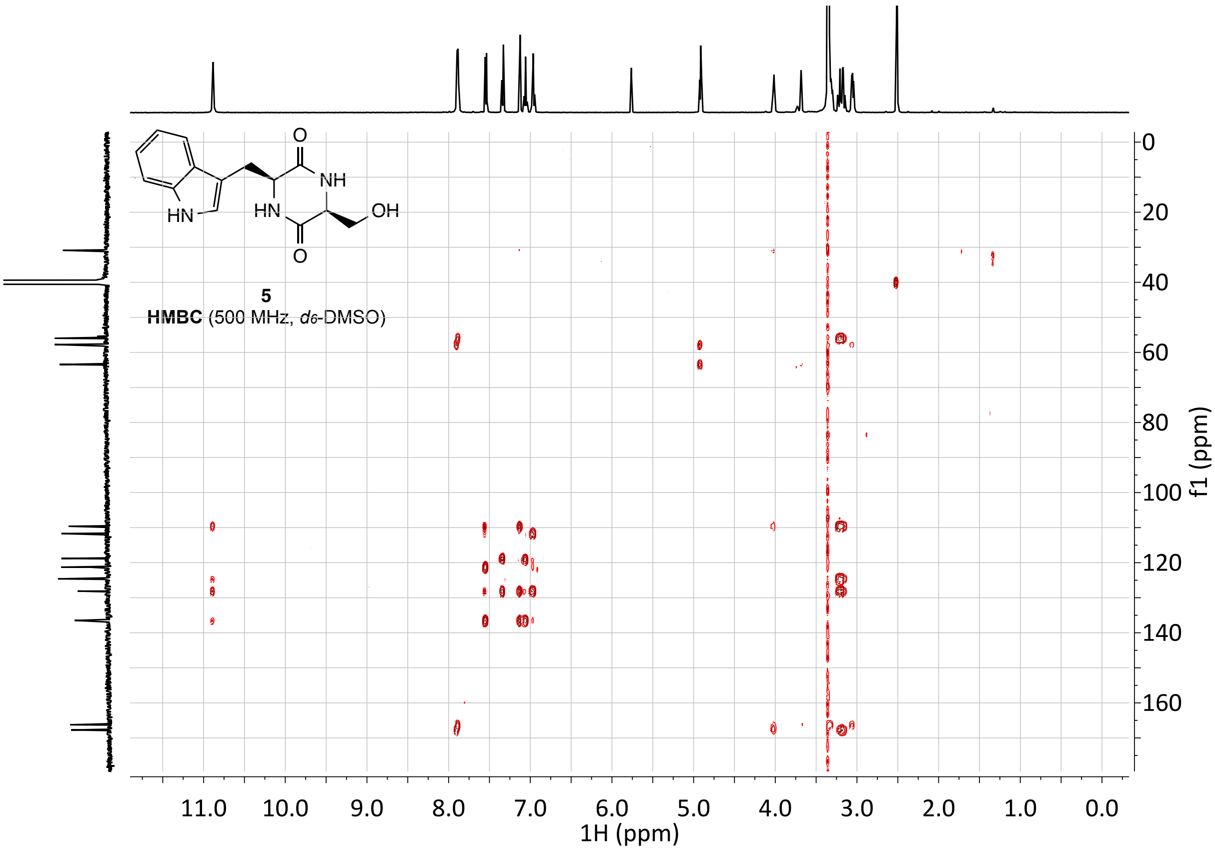
O
NH
HN
HN
OH
O
5
HMBC (500 MHz, d6-DMSO)
1H (ppm)
f1 (ppm)

Supplementary References

1 Malde, A. K. *et al.* An Automated Force Field Topology Builder (ATB) and Repository: Version 1.0. *J Chem Theory Comput* **7**, 4026-4037 (2011). https://doi.org/10.1021/ct200196m

2 Morris, G. M. *et al.* AutoDock4 and AutoDockTools4: Automated docking with selective receptor flexibility. *J Comput Chem* **30**, 2785-2791 (2009). https://doi.org/10.1002/jcc.21256

3 Saha, C. K., Sanches Pires, R., Brolin, H., Delannoy, M. & Atkinson, G. C. FlaGs and webFlaGs: discovering novel biology through the analysis of gene neighbourhood conservation. *Bioinformatics* **37**, 1312-1314 (2021). https://doi.org/10.1093/bioinformatics/btaa788

4 Andreas, M. P. & Giessen, T. W. Cyclodipeptide oxidase is an enzyme filament. *Nat Commun* **15**, 3574 (2024). https://doi.org/10.1038/s41467-024-48030-9

5 Kobori, T. *et al.* Structure and site-directed mutagenesis of a flavoprotein from Escherichia coli that reduces nitrocompounds: alteration of pyridine nucleotide binding by a single amino acid substitution. *J Biol Chem* **276**, 2816-2823 (2001). https://doi.org/10.1074/jbc.M002617200

6 Fulmer, G. R. *et al.* NMR Chemical Shifts of Trace Impurities: Common Laboratory Solvents, Organics, and Gases in Deuterated Solvents Relevant to the Organometallic Chemist. *Organometallics* **29**, 2176-2179 (2010). https://doi.org/10.1021/om100106e

7 Han, Z. Z., Dong, T., Ming, X. X., Kuang, F. & Zhang, C. P. Synthesis and Biological Evaluation of CF(3) Se-Substituted alpha-Amino Acid Derivatives. *ChemMedChem* **16**, 3177-3180 (2021). https://doi.org/10.1002/cmdc.202100451

8 Laroche, B., Tang, X., Archer, G., Di Sanza, R. & Melchiorre, P. Photochemical Chemoselective Alkylation of Tryptophan-Containing Peptides. *Org Lett* **23**, 285-289 (2021). https://doi.org/10.1021/acs.orglett.0c03735

9 Tullberg, M., Grøtli, M. & Luthman, K. Efficient synthesis of 2,5-diketopiperazines using microwave assisted heating. *Tetrahedron* **62**, 7484-7491 (2006). https://doi.org/https://doi.org/10.1016/j.tet.2006.05.010
