# Supplementary material for: Broad substrate scope C-C oxidation in cyclodipeptides catalysed by a flavin-dependent filament
Source: Nat Commun. 2025 Jan 24;16:995. doi: 10.1038/s41467-025-56127-y (PMC11760959; doi:10.1038/s41467-025-56127-y)
Supplement: Supplementary file 2 — Reporting Summary [file 41467_2025_56127_MOESM2_ESM.pdf]

nature portfolio

Corresponding author(s): Clarissa Melo Czekster
Ramasubramanian Sundaramoorthy –

Last updated by author(s): 19/12/2024

Reporting Summary

Nature Portfolio wishes to improve the reproducibility of the work that we publish. This form provides structure for consistency and transparency in reporting. For further information on Nature Portfolio policies, see our Editorial Policies and the Editorial Policy Checklist.

Statistics

For all statistical analyses, confirm that the following items are present in the figure legend, table legend, main text, or Methods section.

n/a	Confirmed	
☐	☒	The exact sample size (*n*) for each experimental group/condition, given as a discrete number and unit of measurement
☐	☒	A statement on whether measurements were taken from distinct samples or whether the same sample was measured repeatedly
☒	☐	The statistical test(s) used AND whether they are one- or two-sided<br>*Only common tests should be described solely by name; describe more complex techniques in the Methods section.*
☒	☐	A description of all covariates tested
☒	☐	A description of any assumptions or corrections, such as tests of normality and adjustment for multiple comparisons
☐	☒	A full description of the statistical parameters including central tendency (e.g. means) or other basic estimates (e.g. regression coefficient) AND variation (e.g. standard deviation) or associated estimates of uncertainty (e.g. confidence intervals)
☒	☐	For null hypothesis testing, the test statistic (e.g. *F*, *t*, *r*) with confidence intervals, effect sizes, degrees of freedom and *P* value noted<br>*Give P values as exact values whenever suitable.*
☒	☐	For Bayesian analysis, information on the choice of priors and Markov chain Monte Carlo settings
☒	☐	For hierarchical and complex designs, identification of the appropriate level for tests and full reporting of outcomes
☒	☐	Estimates of effect sizes (e.g. Cohen's *d*, Pearson's *r*), indicating how they were calculated

Our web collection on statistics for biologists contains articles on many of the points above.

Software and code

Policy information about availability of computer code

Data collection	Software from instrument manufacturer for mass spectrometry (Waters and Sciex), plate reader (BMG labtech), stopped flow (Applied Photophysics) were used
Data analysis	Graphpad prism, Kintek Global Explorer, ChimeraX, Pymol, Coot, Cryosparc, ISOLDE

For manuscripts utilizing custom algorithms or software that are central to the research but not yet described in published literature, software must be made available to editors and reviewers. We strongly encourage code deposition in a community repository (e.g. GitHub). See the Nature Portfolio guidelines for submitting code & software for further information.

Data

Policy information about availability of data

All manuscripts must include a data availability statement. This statement should provide the following information, where applicable:

- Accession codes, unique identifiers, or web links for publicly available datasets
- A description of any restrictions on data availability
- For clinical datasets or third party data, please ensure that the statement adheres to our policy

Manuscript contains the statement as below:
Structure for Ndas1146 (Uniprot: D7B1W6) and Ndas1147 (Uniprot: D7B1W7) was updated to the pdb (accession code 9EXV) and EMDB (accession code EMD-50049). Data for kinetic assays is available in the supporting information file, as well as NMR spectra and spectra for determination of extinction coefficients

used. Raw small molecule mass spectrometry data is available on the Figshare project "Broad substrate scope C-C oxidation in cyclodipeptides catalysed by a flavin-dependent filament" (https://doi.org/10.6084/m9.figshare.25556850). The mass spectrometry proteomics data demonstrating covalent attachment of FMN cofactor have been deposited to the ProteomeXchange Consortium via the PRIDE partner repository (https://doi.org/10.6084/m9.figshare.25556850). The EPR spectroscopic data supporting this publication can be accessed at https://doi.org/10.17630/2e946d01-0753-45ad-aaf3-87572eef9ec0. Data generated in this study are provided in the Supplementary Information/Source Data file. The Supporting Information file includes additional methods, supporting Figures and Tables. Supplementary Table 3 contains information about the cryo-EM structure deposited here.

Research involving human participants, their data, or biological material

Policy information about studies with human participants or human data. See also policy information about sex, gender (identity/presentation), and sexual orientation and race, ethnicity and racism.

Reporting on sex and gender	N/A
Reporting on race, ethnicity, or other socially relevant groupings	N/A
Population characteristics	N/A
Recruitment	N/A
Ethics oversight	N/A

Note that full information on the approval of the study protocol must also be provided in the manuscript.

Field-specific reporting

Please select the one below that is the best fit for your research. If you are not sure, read the appropriate sections before making your selection.

☒ Life sciences ☐ Behavioural & social sciences ☐ Ecological, evolutionary & environmental sciences

For a reference copy of the document with all sections, see nature.com/documents/nr-reporting-summary-flat.pdf

Life sciences study design

All studies must disclose on these points even when the disclosure is negative.

Sample size	All activity assays were done in triplicate
Data exclusions	Only clear outliers were excluded, and this was generally avoided by repeating measurements with new samples.
Replication	All findings could be replicated as assays were carried out in many different days with the repetition of controls to ensure reproducibility. No data could not be replicated.
Randomization	N/A
Blinding	Not possible as we must know identity of proteins and chemicals under use to adjust concentrations and experimental parameters for data acquisition.

Reporting for specific materials, systems and methods

We require information from authors about some types of materials, experimental systems and methods used in many studies. Here, indicate whether each material, system or method listed is relevant to your study. If you are not sure if a list item applies to your research, read the appropriate section before selecting a response.

Materials & experimental systems

n/a	Involved in the study
☒	☐ Antibodies
☒	☐ Eukaryotic cell lines
☒	☐ Palaeontology and archaeology
☒	☐ Animals and other organisms
☒	☐ Clinical data
☒	☐ Dual use research of concern
☒	☐ Plants

Methods

n/a	Involved in the study
☒	☐ ChIP-seq
☒	☐ Flow cytometry
☒	☐ MRI-based neuroimaging

Plants

Seed stocks	N/A
Novel plant genotypes	N/A
Authentication	N/A
